# Correction: Significance of vertical transmission of arboviruses in mosquito-borne disease epidemiology

**DOI:** 10.1186/s13071-025-06864-2

**Published:** 2025-06-18

**Authors:** Oliver Chinonso Mbaoma, Stephanie Margarete Thomas, Carl Beierkuhnlein

**Affiliations:** 1 Department of Biogeography, University of Bayreuth, Bayreuth, Germany; 2 Center of Ecology and Environmental Research, BayCEER, University of Bayreuth, Bayreuth, Germany

**Correction: Parasites & Vectors (2025) 18:137** 10.1186/s13071-025-06761-8

Following publication of the article, the authors flagged miscellaneous errors. The errors have since been corrected in [1]. See the additional file linked to this erratum for the details of the corrections that have been made.

The journal and the authors thank you for reading this erratum and apologize for any inconvenience caused.

## Supplementary Information

Supplementary material 1.
