# Supplementary material for: Significance of vertical transmission of arboviruses in mosquito-borne disease epidemiology
Source: Parasit Vectors. 2025 Apr 9;18:137. doi: 10.1186/s13071-025-06761-8 (PMC11983947; doi:10.1186/s13071-025-06761-8)
Supplement: Supplementary file 1 — Additional file 1. [file 13071_2025_6761_MOESM1_ESM.pdf]

Supplementary Material

1. **Summary Table**

Table S1: Details extracted from studies providing brief details on mosquito species, arbovirus genus, and vertical transmission effici ency.

Mosquito Species	Arbovirus genus	Location	VT Efficiency	References
**Modeling Studies**				
Aedes	DENV	Cuba		[1]
Aedes, Culex	RVFV			[2]
Aedes	DENV	China		[3]
Ae. aegypti	ZIKV			[4]
Aedes	DENV			[5]
Aedes	DENV			[6]
Aedes	ZIKV			[7]
Aedes	DENV			[8]
Aedes	CHIKV	India		[9]
Aedes	DENV	Saudi Arabia		[10]
Ae. aegypti	DENV			[11]
Ae. aegypti	DENV	Brazil		[12]
Ae. aegypti	DENV	Thailand		[13]
Ae. aegypti	DENV	Peru		[14]
Ae. albopictus	DENV	China		[15]
Aedes	DENV			[16]
Aedes	DENV			[17]
Aedes	RVFV			[18]
Culex	WNV	United States		[19]
Ae. vexans, Ae. ochraceaus, Cx. poicilipes	RVFV	East Africa		[20]
Ae. vigilax, Cx. annulirostris, Ae. camptorhynchus, Cx. globocoxitus	RRV	Australia		[21]
Aedes, Cx. and Eretmapodites)	RVFV	France		[22]
Cx.pipiens	WNV			[23]
Cx.pipiens	WNV			[24]
**Laboratory Studies**				
Ae. africanus	YFV	Uganda	ND	[25]

Ae. aegypti	YFV			[26]
Cx. tritaeniorhynchus	WNV	India	ND	[27]
Ae. aegypti	YFV		Minimum filial infection 1:596	[28]
Ae. aegypti	DENV	Vietnam, Guadeloupe (France) and Nigeria	Minimum filial infection 0.3-1.2%	[29]
Ae. Aegypti., Ae. albopictus	DENV			[30]
Ae. mediovittatus	DENV	Puerto Rio		[31]
Multiple Aedes Species	DENV	Thailand	Filial infection rates *Ae. cooki* 6.7% *Ae. Polynesinesis* 4.6%	[32]
Ae. Aegypti., Ae. albopictus	CHIKV	Thailand	ND	[33]
Ae. Albopictus	DENV	Brazil	MIR 1:84 (larvae) 1:311(adult female)	[34]
Ae. albopictus	DENV	Jamaica	Filial infection rate 0.5 to 2.9% (among strains) 1.4 to 17.4% (within strains)	[35]
Multiple mosquito species	RVFV, EEE, CHIKV			[36]
Ae. Albopictus, Ae. Aegypti, Cx. tritaeniorhynchus	WNV		Minimal filial infection rate *Ae. Albopictus* 1:124 to 1:138 *Ae. Aegypti* 1:62 to 1:172 *Cx. tritaeniorhynchus* 1:325 to 1:859	[37]
Cx. pipiens	WNV	United States		[38]
Cx. vishuni	WNV		5.5%	[39]
Ae. aegypti	DENV			[40]
Cx. Pipiens	WNV	United States	MIR 1.4-2.1 18 and 20 degrees C.	[41]
Ae. aegypti	DENV	India		[42]
Ae. Aegypti., Ae. albopictus	CHIKV	India	ND	[43]

Cx. pipiens pipiens., Cx. quinquefasciatus Cx. tarsalis	WNV	United States	MIR<br>*Cx. Quinquefasciatus*<br>3.0<br>*Cx. Tarsalis*<br>6.9	[44]
Ae. albopictus	DENV	Brazil	39.1%	[45]
Ae. Albopictus	CHIKV	La Reunion Island	ND	[46]
Ae. albopictus	CHIKV	Italy	ND	[47]
Cx. pipiens molestus, Cx. quinquefasciatus	WNV	Argentina	MIR<br>*Cx. pipiens molestus*<br>1.19	[48]
Cx. pipiens molestus	WNV	United States	MIR<br>0.6	[49]
Ae. aegypti	DENV	Indonesia		[50]
Cx. gelidus	WNV	India	ND	[51]
Cx. quinquefasciatus	WNV	India	ND	[52]
Ae. aegypti	CHIKV	Malaysia	ND	[53]
Ae. aegypti,<br>*Ae. albopictus*	DENV	United States		[54]
Ae. aegypti	ZIKV	China	MIR<br>2.13	[55]
Ae. aegypti	DENV, CHIKV, ZIKV	India	ND	[56]
Ae. albopictus,<br>*Ae. caspius*	ZIKV	Spain	Filial infection rate<br>1:14	[57]
Ae. aegypti	ZIKV	China	MIR<br>1:14.29	[58]
Ae. aegypti	ZIKV	Singapore	Filial infection rate<br>8.5-66%	[59]
Ae. aegypti,<br>*Ae. albopictus*	DENV	Vietnam	Percentage<br>*Ae. aegypti*<br>2.34%	[60]
Ae. albopictus	ZIKV	China		[61]
Ae. albopictus	ZIKV			[62]
Ae. albopictus	WNV	China	MIR<br>1:650	[63]
Ae. albopictus	ZIKV	China		[64]
Ae. aegypti	ZIKV	Brazil		[65]

Ae. aegypti	DENV	United States		[66]
Ae. aegypti	CHIKV	India	FIR<br>Lavre: of 28.2<br>Adult: 20.2	[67]
Ae. aegypti	ZIKV	United States	MTR<br>6.5	[68]
Ae. aegypti	DENV	United States	Percentage 19/68 (27.9%)	[69]
Ae. albopictus	ZIKV	China	FIR<br>Lavre:2.06%<br>Adult:1.87%	[70]
Ae. albopictus	ZIKV	Spain		[71]
Ae. albopictus	ZIKV	South Korea	MIR<br>Egg:1.0<br>Larvae:1.3<br>Adult:6.7	[72]
Ae. albopictus	CHIKV	Brazil/United States	MIR<br>0.76	[73]
Ae. vexans	WNV	United States	Percentage<br>1.1%	[74]
Cx. pipiens	WNV	United States	MIR<br>0.52	[75]
Ae. aegypti and *Ae. albopictus*	CHIKV	Thailand	MIR<br>*Ae. aegypti*<br>Larvae:8.33<br>Adult:10<br>*Ae. albopictus*<br>18.33: Larvae<br>21.67: Adults	[76]
Ae. aegypti and *Ae. albopictus*	ZIKV	Spain	ND	[77]
Ae. aegypti and *Ae. albopictus*	ZIKV	Puerto Rico	FIR<br>*Ae. aegypti*<br>1.1-3.2%<br>*Ae. albopictus*<br>0-0.3%	[78]
Cx pipiens	WNV	Italy	ND	[79]
Cx. quinquefasciatus	WNV	Galápagos Island	MIR<br>3.7	[80]

Ae. aegypti and *Ae. albopictus*	DENV	United States	Percentage<br>*Ae. albopictus*<br>11.11%<br>*Ae aegypti*<br>8.33%	[81]
Ae. aegypti, Ae. albopictus and Cx. quinquefasciatus	ZIKV	Thailand	*Cx. quinquefasciatus*<br>1:66<br>*Ae. aegypti*<br>1:1200	[82]
Ae. aegypti	ZIKV	Mexico	Percentage<br>40%	[83]
Ae. aegypti	YFV	Senegal	Percentage<br>0.97%	[84]
Ae. aegypti and *Ae. albopictus*	ZIKV	Venezuela	FIR<br>*Ae. aegypti*<br>1:290	[85]
**Field Studies**				
Ae. aegypti	DENV	Myanmar	Minimum filial infection<br>1:2067	[86]
Ae. Aegypti	DENV	Trinidad		[87]
Ae. aegypti	DENV	Thailand	ND	[88]
Ae. camptorhynchus	RRV, SINV	Australia		[89]
Ae. Aegypti,<br>*Ae. furcifer, Ae. metallicus and Ae. luteocephalus*	YFV	Senegal	True infection rate<br>1.4% and 0.5%<br>Female and male	[90]
Ae.aegypti,	DENV	Colombia	ND	[91]
Cx. erythrothorax	WNV	United States		[92]
Ae. albopictus	DENV	India		[93]
Ae. albopictus	DENV	India	MIR<br>2.2	[94]
Ae. aegypti	DENV	India	MIR<br>28	[95]
Cx. pipiens	WNV	United States		[96]
Ae. aegypti	DENV	Brazil	ND	[97]
Ae. albopictus	DENV	Brazil	MIR<br>1:36.45	[98]
Ae. aegypti ,<br>*Ae. albopictus*	DENV	Indonesia	Percentage<br>*Ae. aegypti*	[99]

			0.48%-8.77%	
Multiple Aedes and Culex species	WNV	United States		[100]
Cx. quinquefasciatus	SLEV	Argentina	MIR<br>3.4	[101]
Ae. aegypti	DENV	Indonesia	Percentage<br>20%	[102]
Ae. albopictus	DENV	Thailand	MIR<br>12.3	[103]
Ae. aegypti ,<br>*Ae. albopictus*	DENV	Malaysia	Percentage<br>6.3%	[104]
Ae. aegypti ,<br>*Ae. albopictus*	DENV	Pakistan		[105]
Ae. aegypti	DENV	Philippines	MIR<br>48.2	[106]
Ae. aegypti ,<br>*Ae. albopictus*	DENV	India	MIR<br>*Ae. aegypti*<br>10.87, 11.3<br>Female/Male	[107]
Ae. aegypti	DENV	India	MIR<br>5.8	[108]
Ae. aegypti	DENV	Cape Verde	ND	[109]
Ae. japonicus,<br>*Ae. albopictus, Ae. triseriatus*	La Crosse Virus	United States	MIR<br>*Ae. triseriatus*<br>2.72<br>*Ae. albopictus*<br>3.01<br>*Ae. japonicus*<br>0.63	[110]
Multiple mosquito species	SINV	Sweden		[111]
Aedes	DENV	India	1.09%	[112]
Ae. aegypti ,<br>*Ae. albopictus*	DENV	Pakistan	MIR<br>*Ae. aegypti*<br>13.3<br>*Ae. albopictus*<br>11.1	[113]
Ae. albopictus	ZIKV	Brazil		[114]
Ae. aegypti ,<br>*Ae. albopictus*	CHIKV, DENV, ZIKV	Brazil	MIR<br>*Ae. aegypti*	[115]

			0.45 CHIKV, ZIKV	
Multiple mosquito species	Multiple arboviruses	Kenya		[116]
Ae. aegypti	CHIKV	Mexico		[117]
Ae. albopictus	DENV	Japan	ND	[118]
Ae. aegypti	DENV	Mexico		[119]
Ae. aegypti	ZIKV	Mexico	MIR 2.8, 6.9 June, November	[120]
Ae. aegypti , *Ae. albopictus*	ZIKV	Indonesia		[121]
Ae. aegypti , *Ae. albopictus*	ZIKV	Colombia		[122]
Multiple mosquito species	ZIKV	Senegal		[123]
Ae. albopictus	DENV	Malaysia	MIR 47.6, 12.5,11.8,12.5	[124]
Ae. aegypti , *Ae. albopictus*	DENV	Sri Lanka	*Ae. aegypti* 8.1% *Ae. albopictus* 9.8%	[125]
Ae. aegypti	DENV	Indonesia	5.6%	[126]
Ae. aegypti , *Ae. albopictus*	DENV	Indonesia		[127]
Ae. aegypti	DENV, CHIKV, ZIKV	Brazil		[128]
Ae. albopictus	DENV	Brazil	MIR 0.61	[129]
Ae. aegypti	ZIKV	Brazil		[130]
Ae. aegypti	CHIKV	India	MIR 4.2-33.3	[131]
Ae. aegypti	DENV	Thailand	MIR 0-24.4	[132]
Ae. aegypti	DENV	Brazil	ND	[133]
Ae. aegypti	DENV	Mexico		[134]
Ae. aegypti	CHIKV, DENV, ZIKV	Kenya		[135]
Ae. aegypti, *Ae. albopictus*	DENV	Mexico		[136]
Ae. aegypti	CHIKV, DENV	France		[137]
Ae aegypti	DENV	Mexico	MIR 0.32	[138]
Ae. aegypti	CHIKV	Brazil	MIR	[139]

	DENV<br>ZIKV		ZIKV:0.45<br>CHIKV:0.45:	
Ae. aegypti	DENV	Mexico	DENV:22.32	[140]
Ae. aegypti and *Ae. albopictus*	Multiple Arboviruses	Brazil		[141]
Ae. aegypti, Ae. albopictus	DENV	Brazil	MIR<br>*Ae. aegypti*<br>3.37	[142]
Multiple mosquito species	DENV	Brazil		[143]
Cx.pipiens, Cs.annulata, An.Maculipennis, An.plumbeus	WNV	Austria		[144]
Ae. aegypti	DENV	Brazil	ND	[145]
Ae. aegypti	DENV	Bolivia	MIR<br>DENV:1.01	[146]
Cx.pipiens	WNV	United States.	FIR<br>WNV:2	[147]
Ae. aegypti	DENV	Brazil	MIR<br>DENV:17.7	[148]
Ae. aegypti, *Ae. albopictus*	DENV	Brazil	MIR<br>*Ae. aegypti*:<br>0.5<br>*Ae. albopictus*<br>9.4	[149]
Ae. aegypti	DENV	Brazil	ND	[150]
Ae. albopictus, Hg. leucocelaenus	ZIKV, YFV	Brazil		[151]
Ae. aegypti	DENV YFV, WNV, SLEV	Brazil	MIR<br>DENV:10.5	[152]
Ae. aegypti	DENV	Colombia		[153]
Ae. aegypti	DENV, CHIKV, ZIKV	Brazil		[154]
Ae. aegypti	DENV	Cuba	MIR<br>DENV: 11.1	[155]
Ae. aegypti, *Ae. albopictus*	DENV	Brazil		[156]
Ae. aegypti, *Ae. albopictus*	DENV	Brazil		[157]
Ae. aegypti	DENV, CHIKV, ZIKV	Brazil	Percentage<br>DENV:25%<br>CHIKV:15%	[158]
Ae. aegypti, *Ae. albopictus*	DENV	Brazil	Percentage	[159]

			Ae. aegypti:<br>37.4%<br>*Ae. albopictus*<br>50%	
Ae. aegypti	DENV	Cuba	MIR<br>9.02	[160]
Ae. aegypti	DENV	Cuba	MIR<br>RTPCR :14.81<br>RT-LAMP: 33.33	[161]
Ae. aegypti	DENV	Brazil	MIR<br>DENV:0.18	[162]
Ae. albopictus	DENV	Cuba	MIR<br>8.88	[163]
Ae. aegypti	DENV	Mexico	Percentage<br>0.9%	[164]
Ae. aegypti, *Ae. albopictus*	DENV	Mexico	MIR<br>*Ae. aegypti*<br>DENV:2.5	[165]
Cx.pipiens, An. maculipennis, Cs. annulata	WNV	Czech Republic		[166]
Ae. aegypti	DENV	French Guiana		[167]
Ae. aegypti	DENV	Brazil		[168]
Ae. aegypti	DENV	Thailand	Percentage<br>49%	[169]
Ae. aegypti	DENV	Colombia		[170]
Ae. aegypti, Ae. albopictus	DENV	Indonesia	*Ae. aegypti*<br>18.3%<br>*Ae. albopictus*<br>16.1%	[171]
Ae. albopictus	CHIKV	India		[172]
**Field and Lab Studies**				
Cx.pipiens, Cx. torrentium	SINV	Sweden	Percentage<br>*Cx.pipiens*<br>23%	[173]
Cx.pipiens, *Cx. tarsalis*	WNV	United States	MIR<br>*Cx.pipiens*<br>3.5	[174]

Cx. tarsalis, Cx. quinquefasciatus, Cx. stigmatosoma	WNV	United States	FIR<br>*Cx.tarsalis*<br>8.1	[175]

*DENV - Dengue Virus

*ZIKV - Zika Virus

*WNV - West Nile Virus

*RVFV - Rift Valley Fever Virus

*SINV - Sindbis Virus

*RRV - Ross River Virus

*MAYV - Mayaro Virus

*VT - Vertical Transmission

*HT - Horizontal Transmission

*MIR - Minimum Infection Rate

*FIR - Filial Infection Rate

2. Figure

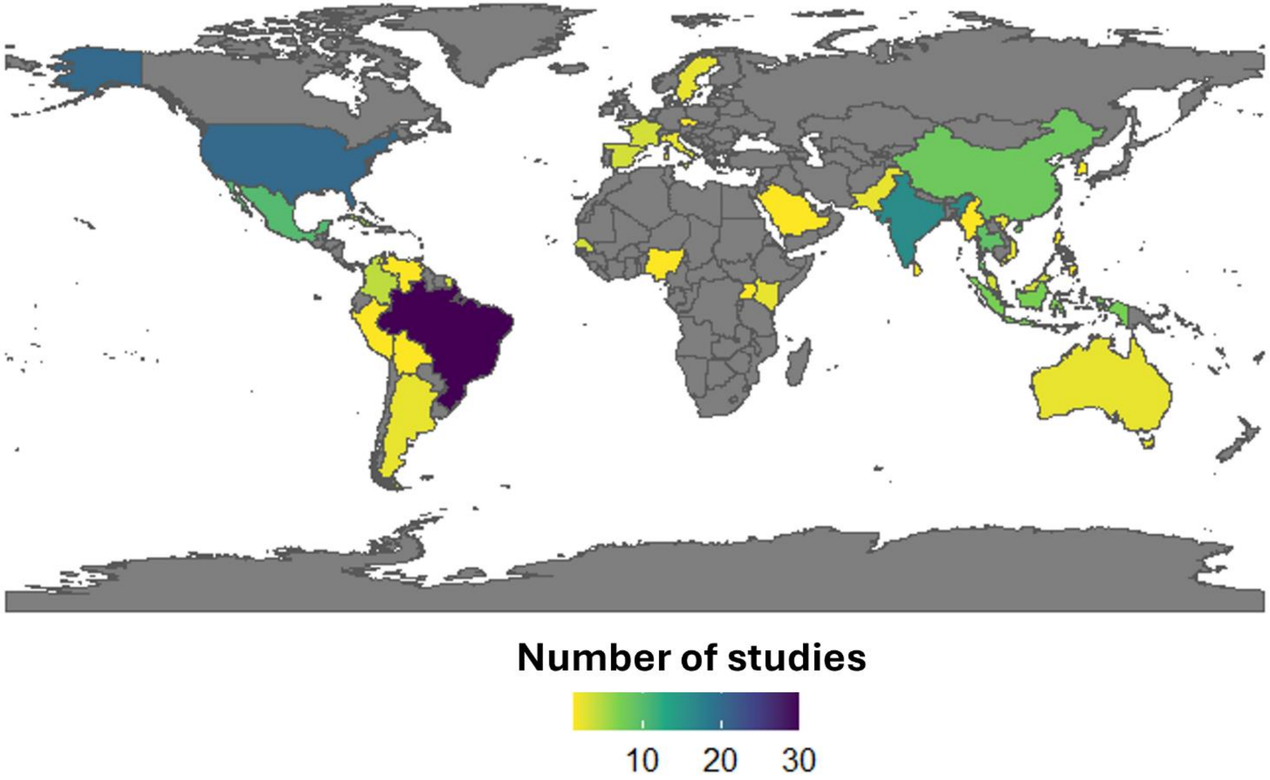

Fig. S1 Global distribution of studies on vertical transmission of arboviruses in mosquito population extracted from literature used for the study.

References.

1. Barrios, J., Piétrus, A., Joya, G., Marrero, A. and de Arazoza, H., 2013. A differential inclusion approach for modeling and analysis of dynamical systems under uncertainty: Application to dengue disease transmission. Soft Computing, 17, pp.239-253.

2. Pedro, S.A., Abelman, S., Ndjomatchoua, F.T., Sang, R. and Tonnang, H.E., 2014. Stability, bifurcation and chaos analysis of vector-borne disease model with application to rift valley fever. PloS one, 9(10), p.e108172.

3. Zou, L., Chen, J., Feng, X. and Ruan, S., 2018. Analysis of a dengue model with vertical transmission and application to the 2014 dengue outbreak in Guangdong Province, China. Bulletin of mathematical biology, 80, pp.2633-2651.

4. Olawoyin, O. and Kribs, C., 2018. Effects of multiple transmission pathways on Zika dynamics. Infectious Disease Modelling, 3 , pp.331-344.

5. Li, M. and Zhao, H., 2021. Dynamics of a dengue fever model with vertical transmission and time periodic in spatially heterog eneous environments. Mathematical Methods in the Applied Sciences, 44(14), pp.11350-11375.

6. Abidemi, A., Ahmad, R. and Aziz, N.A.B., 2021. Assessing the roles of human movement and vector vertical transmission on deng ue fever spread and control in connected patches: from modelling to simulation. The European Physical Journal Plus, 136(11), p.1192.

7. Yuan, X., Lou, Y., He, D., Wang, J. and Gao, D., 2021. A Zika endemic model for the contribution of multiple transmission rou tes. Bulletin of Mathematical Biology, 83, pp.1-28.

8. Li, M. and Zhao, H., 2022. Dynamics of a reaction–diffusion dengue fever model with incubation periods and vertical transmission in heterogeneous environments. Journal of Applied Mathematics and Computing, 68(6), pp.3673-3703.

9. Wang, Y., Li, Y., Liu, L. and Liu, X., 2022. A periodic Chikungunya model with virus mutation and transovarial transmission. Chaos, Solitons & Fractals, 158, p.112002.

10. Alsheri, A.S., 2024. Impact of Seasonality and Vertical Transmission on Mosquito Population in the Dynamics of Dengue Disease. Contemporary Mathematics, pp.546-560.

11. Abdullah S, Seadawy A, Jun W. New mathematical model of vertical transmission and cure of vector-borne diseases and its numerical simulation. Adv Differ Equ. 2018;2018:1-15. https://doi.org/10.1186/s13662-018-1516-z.

12. Alves LD, Lana RM, Coelho FC. A framework for weather-driven dengue virus transmission dynamics in different Brazilian regions. Int J Environ Res Public Health. 2021;18(18):9493. https://doi.org/10.3390/ijerph18189493.

13. Taghikhani R, Gumel AB. Mathematics of dengue transmission dynamics: Roles of vector vertical transmission and temperature fluctuations. Infect Dis Model. 2018;3:266-92. https://doi.org/10.1016/j.idm.2018.09.003.

14. Murillo D, Murillo A, Lee S. The role of VT in the control of dengue fever. Int J Environ Res Public Health. 2019;16(5):803. https://doi.org/10.3390/ijerph16050803.

15. Cheng Q, Jing Q, Spear RC, Marshall JM, Yang Z, Gong P. Climate and the timing of imported cases as determinants of the dengu e outbreak in Guangzhou, 2014: Evidence from a mathematical model. PLoS Negl Trop Dis. 2016;10(2):e0004417. https://doi.org/10.1371/journal.pntd.0004417.

16. Blayneh KW. Uniform persistence and backward bifurcation of vertically transmitted vector-borne diseases. Res Math. 2023;10(1):2264581. https://doi.org/10.1080/27684830.2023.2264581.

17. Aliyu AI, Inc M, Yusuf A, Baleanu D. A fractional model of vertical transmission and cure of vector-borne diseases pertaining to the Atangana–Baleanu fractional derivatives. Chaos Solitons Fractals. 2018;116:268-77. https://doi.org/10.1016/j.chaos.2018.09.043.

18. Chitnis N, Hyman JM, Manore CA. Modelling vertical transmission in vector-borne diseases with applications to RVFV. J Biol Dyn. 2013;7(1):11-40. https://doi.org/10.1080/17513758.2012.733427.

19. Wang FB, Wu R, Zhao XQ. A West Nile virus transmission model with periodic incubation periods. SIAM J Appl Dyn Syst. 2019;18(3):1498-535. https://doi.org/10.1137/18M1236162.

20. Favier C, Chalvet-Monfray K, Sabatier P, Lancelot R, Fontenille D, Dubois MA. Rift Valley fever in West Africa: the role of s pace in endemicity. Trop Med Int Health. 2006;11(12):1878-88. https://doi.org/10.1111/j.1365-3156.2006.01746.x.

21. Koolhof IS, Beeton N, Bettiol S, Charleston M, Firestone SM, Gibney K, et al. Testing the intrinsic mechanisms driving the dy namics of Ross River Virus across Australia. PLoS Pathog. 2024;20(2):e1011944. https://doi.org/10.1371/journal.ppat.1011944.

22. Cavalerie L, Charron MV, Ezanno P, Dommergues L, Zumbo B, Cardinale E. A stochastic model to study Rift Valley fever persiste nce with different seasonal patterns of vector abundance: new insights on the endemicity in the tropical island of Mayotte. PLoS On e. 2015;10(7):e0130838. https://doi.org/10.1371/journal.pone.0130838.

23. Fan G, Liu J, Van den Driessche P, Wu J, Zhu H. The impact of maturation delay of mosquitoes on the transmission of West Nile virus. Math Biosci. 2010;228(2):119-26. https://doi.org/10.1016/j.mbs.2010.08.010

24. Moschini P, Bisanzio D, Pugliese A. A seasonal model for West Nile virus. Math Model Nat Phenom. 2017;12(2):58-83. https://doi.org/10.1051/mmnp/201712205.

25. Gillett, J.D., Ross, R.W., Dick, G.W.A., Haddow, A.J. and Hewitt, L.E., 1950. Experiments to test the possibility of transova rial transmission of yellow fever virus in the mosquito Aedes (Stegomyia) africanus Theobald. *Annals of Tropical Medicine & Parasitology*, *44*(4), pp.342-350.

26. Aitken, T.H., Tesh, R.B., Beaty, B.J. and Rosen, L., 1979. Transovarial transmission of yellow fever virus by mosquitoes (Aed es aegypti). The American journal of tropical medicine and hygiene, 28(1), pp.119-121.

27. Hayes, C.G., Basit, A., Bagar, S. and Akhter, R., 1980. Vector competence of Culex tritaeniorhynchus (diptera: Culicidae) for west nile virus. Journal of medical entomology, 17(2), pp.172-177.

28. Beaty, B.J., Tesh, R.B. and Aitken, T.H., 1980. Transovarial transmission of yellow fever virus in Stegomyia mosquitoes. The American journal of tropical medicine and hygiene, 29(1), pp.125-132.

29. Jousset, F.X., 1981, July. Geographic Aedes aegypti strains and dengue-2 virus: susceptibility, ability to transmit to vertebrate and transovarial transmission. In Annales de l'Institut Pasteur/Virologie (Vol. 132, No. 3, pp. 357-370). Elsevier Masson.

30. Rosen, L., Shroyer, D.A., Tesh, R.B., Freier, J.E. and Lien, J.C., 1983. Transovarial transmission of dengue viruses by mosqu itoes: Aedes albopictus and Aedes aegypti. The American journal of tropical medicine and hygiene, 32(5), pp.1108-1119.

31. Gubler, D.J., Novak, R.J., Vergne, E., Colon, N.A., Velez, M. and Fowler, J., 1985. Aedes (Gymnometopa) mediovittatus (Diptera: Culicidae), a potential maintenance vector of dengue viruses in Puerto Rico. Journal of medical entomology, 22(5), pp.469-475.

32. Freier, J.E. and Rosen, L., 1987. Vertical transmission of dengue viruses by mosquitoes of the Aedes scutellaris group. The American journal of tropical medicine and hygiene, 37(3), pp.640-647.

33. Mourya, D.T., 1987. Absence of transovarial transmission of Chikungunya virus in Aedes aegypti & Ae. albopictus mosquitoes.

34. Mitchell, C.J. and Miller, B.R., 1990. Vertical transmission of dengue viruses by strains of Aedes albopictus recently introd uced into Brazil. Journal of the American Mosquito Control Association, 6(2), pp.251-253.

35. Bosio, C.F., Thomas, R.E., Grimstad, P.R. and Rai, K.S., 1992. Variation in the efficiency of vertical transmission of dengue-1 virus by strains of Aedes albopictus (Diptera: Culicidae). Journal of medical entomology, 29(6), pp.985-989.

36. Turell, M.J., 1992. Virus-dependent mortality in Rift Valley fever, eastern equine encephalomyelitis, and chikungunya virus-inoculated mosquito (Diptera: Culicidae) larvae. Journal of medical entomology, 29(5), pp.792-795.

37. Baqar, S., Hayes, C.G., Murphy, J.R. and Watts, D.M., 1993. Vertical transmission of West Nile virus by Culex and Aedes speci es mosquitoes. The American journal of tropical medicine and hygiene, 48(6), pp.757-762.

38. Turell, M.J., O'Guinn, M.L., Dohm, D.J. and Jones, J.W., 2001. Vector competence of North American mosquitoes (diptera: culic idae) for West Nile virus. *Journal of medical entomology*, *38*(2), pp.130-134.

39. Mishra, A.C. and Mourya, D.T., 2001. Transovarial transmission of West Nile virus in Culex vishnui mosquito. Indian Journal of Medical Research, 114, p.212.

40. Mourya, D.T., Gokhale, M.D., Basu, A., Barde, P.V., Sapkal, G.N., Padbidri, V.S. and Gore, M.M., 2001. Horizontal and vertical transmission of dengue virus type 2 in highly and lowly susceptible strains of Aedes aegypti mosquitoes. Acta virologica, 45(2), pp.67-72.

41. Dohm, D.J., Sardelis, M.R. and Turell, M.J., 2002. Experimental vertical transmission of West Nile virus by Culex pipiens (Diptera: Culicidae). Journal of medical Entomology, 39(4), pp.640-644.

42. Joshi, V., Mourya, D.T. and Sharma, R.C., 2002. Persistence of dengue-3 virus through transovarial transmission passage in successive generations of Aedes aegypti mosquitoes. The American journal of tropical medicine and hygiene, 67(2), pp.158-161.

43. Hundekar, S.L., Thakare, J.P., Gokhale, M.D. and Barde, S.V., 2002. Development of monoclonal antibody based antigen capture ELISA to detect chikungunya virus antigen in mosquuitoes. Indian Journal of Medical Research, 115, p.144.

44. Goddard, L.B., Roth, A.E., Reisen, W.K. and Scott, T.W., 2003. Vertical transmission of west nile virus by three california Culex (Diptera: Culicidae) species. Journal of medical entomology, 40(6), pp.743-746.

45. Castro, M.G.D., Nogueira, R.M.R., Schatzmayr, H.G., Miagostovich, M.P. and Lourenço-de-Oliveira, R., 2004. Dengue virus detection by using reverse transcription-polymerase chain reaction in saliva and progeny of experimentally infected Aedes albopictus from Brazil. Memórias do Instituto Oswaldo Cruz, 99, pp.809-814

46. Vazeille, M., Mousson, L. and Failloux, A.B., 2009. Failure to demonstrate experimental vertical transmission of the epidemic strain of Chikungunya virus in Aedes albopictus from La Réunion Island, Indian Ocean. Memórias do Instituto Oswaldo Cruz, 104, pp.632-635.

47. Bellini, R., Medici, A., Calzolari, M., Bonilauri, P., Cavrini, F., Sambri, V., Angelini, P. and Dottori, M., 2012. Impact of Chikungunya virus on Aedes albopictus females and possibility of vertical transmission using the actors of the 2007 outbreak in Italy. PLoS One, 7(2), p.e28360.

48. Micieli, M.V., Matacchiero, A.C., Muttis, E., Fonseca, D.M., Aliota, M.T. and Kramer, L.D., 2013. Vector competence of Argentine mosquitoes (Diptera: Culicidae) for West Nile virus (Flaviviridae: Flavivirus). Journal of medical entomology, 50(4), pp.853-862.

49. Nelms, B.M., Kothera, L., Thiemann, T., Macedo, P.A., Savage, H.M. and Reisen, W.K., 2013. Phenotypic variation among Culex pipiens complex (Diptera: Culicidae) populations from the Sacramento Valley, California: horizontal and vertical transmission of West Nile virus, diapause potential, autogeny, and host selection. The American Journal of Tropical Medicine and Hygiene, 89(6), p.1168.

50. Satoto, T.B.T., Umniyati, S., Suardipa, A. and Sintorini, M., 2013. Effects of temperature, relative humidity, and DEN-2 virus transovarial infection on viability of Aedes aegypti. Kesmas, 7(7), pp.331-336

51. Sudeep, A.B., Ghodke, Y.S., Gokhale, M.D., George, R.P., Dhaigude, S.D. and Bondre, V.P., 2014. Replication potential and different modes of transmission of West Nile virus in an Indian strain of Culex gelidus Theobald (Diptera: Culicidae). Journal of Vector Borne Diseases, 51(4), pp.333-338.

52. Sudeep, A.B., Mandar, P., Ghodke, Y.K., George, R.P. and Gokhale, M.D., 2015. Vector competence of two Indian populations of Culex quinquefasciatus (Diptera: Culicidae) mosquitoes to three West Nile virus strains. Journal of Vector Borne Diseases, 52(3), pp.185-192.

53. Wong, H.V., Vythilingam, I., Sulaiman, W.Y.W., Lulla, A., Merits, A., Chan, Y.F. and Sam, I.C., 2016. Detection of persistent chikungunya virus RNA but not infectious virus in experimental vertical transmission in Aedes aegypti from Malaysia. The American Journal of Tropical Medicine and Hygiene, 94(1), p.182.

54. Buckner, E.A., Alto, B.W. and Lounibos, L.P., 2016. Larval temperature–food effects on adult mosquito infection and vertical transmission of dengue-1 virus. Journal of Medical Entomology, 53(1), pp.91-98.

55. Li, C.X., Guo, X.X., Deng, Y.Q., Xing, D., Sun, A.J., Liu, Q.M., Wu, Q., Dong, Y.D., Zhang, Y.M., Zhang, H.D. and Cao, W.C., 2017. Vector competence and transovarial transmission of two Aedes aegypti strains to Zika virus. Emerging microbes & infections, 6(1), pp.1-7.

56. Mourya, D.T., Gokhale, M.D., Majumdar, T.D., Yadav, P.D., Kumar, V. and Mavale, M.S., 2018. Experimental Zika virus infection in Aedes aegypti: Susceptibility, transmission & co-infection with dengue & chikungunya viruses. Indian Journal of Medical Research, 147(1), pp.88-96.

57. Gutiérrez-López, R., Bialosuknia, S.M., Ciota, A.T., Montalvo, T., Martínez-de la Puente, J., Gangoso, L., Figuerola, J. and Kramer, L.D., 2019. Vector competence of Aedes caspius and Ae. albopictus mosquitoes for Zika virus, Spain. Emerging infectious diseases, 25(2), p.346.

58. Zhu, C., Jiang, Y., Zhang, Q., Gao, J., Gu, Z., Lan, C., Li, C., Li, C., Dong, Y., Xing, D. and Li, J., 2020. Vertical transmission of zika virus by jiegao and mengding Aedes aegypti (Diptera: Culicidae) strains in Yunnan province in China. Vector-Borne and Zoonotic Diseases, 20(9), pp.664-669

59. Manuel, M., Missé, D. and Pompon, J., 2020. Highly efficient vertical transmission for Zika virus in Aedes aegypti after long extrinsic incubation time. Pathogens, 9(5), p.366.

60. Goncalves, D.D.S., Hue, K.D.T., Thuy, V.T., Tuyet, N.V., Thi, G.N., Thi Thuy, V.H., Xuan, T.H.T., Thi, D.L., Vo, L.T., Le Anh Huy, H. and Van Thuy, N.T., 2020. Assessing the vertical transmission potential of dengue virus in field-reared Aedes aegypti using patient-derived blood meals in Ho Chi Minh City, Vietnam. Parasites & Vectors, 13, pp.1-9.

61. Guo, X., Li, C., Deng, Y., Jiang, Y., Sun, A., Liu, Q., Dong, Y., Xing, D., Cao, W., Qin, C. and Zhao, T., 2020. Vector competence and vertical transmission of Zika virus in Aedes albopictus (Diptera: Culicidae). Vector-Borne and Zoonotic Diseases, 20(5), pp.374-379.

62. Guo, Y., Guo, J. and Li, Y., 2022. Wolbachia w Pip blocks Zika virus transovarial transmission in Aedes albopictus. Microbiology Spectrum, 10(5), pp.e02633-21.

63. Zhang, Y.M., Guo, X.X., Jiang, S.F., Li, C.X., Xing, D., Zhang, H.D., Dong, Y.D. and Zhao, T.Y., 2022. The potential vector competence and overwintering of West Nile virus in vector Aedes albopictus in China. Frontiers in Microbiology, 13, p.888751.

64. Jian, X.Y., Jiang, Y.T., Wang, M., Jia, N., Cai, T., Xing, D., Li, C.X., Zhao, T.Y., Guo, X.X. and Wu, J.H., 2023. Effects of constant temperature and daily fluctuating temperature on the transovarial transmission and life cycle of Aedes albopictus infected with Zika virus. Frontiers in Microbiology, 13, p.1075362.

65. da Encarnação Sá-Guimarães T, Salles TS, Rocha dos Santos C, et al. Route of Zika virus infection in Aedes aegypti by transmission electron microscopy. BMC Microbiol. 2021;21:300. https://doi.org/10.1186/s12866-021-02366-0.

66. Stephenson CJ, iedworth H, Kang S, Lednicky JA, Dinglasan RR. Transmission potential of Floridian Aedes aegypti mosquitoes for DENV virus serotype 4: Implications for estimating local dengue risk. mSphere. 2021;6(4):10-1128. https://doi.org/10.1128/mSphere.00271-21.

67. Agarwal A, Dash PK, Singh AK, Sharma S, Gopalan N, Rao PVL, et al. Evidence of experimental vertical transmission of emerging novel ECSA genotype of Chikungunya Virus in Aedes aegypti. PLoS Negl Trop Dis. 2014;8(7):e2990. https://doi.org/10.1371/journal.pntd.0002990.

68. Comeau G, Zinna RA, Scott T, Ernst K, Walker K, Carrière Y, et al. VT of Zika virus in Aedes aegypti produces potentially infectious progeny. Am J Trop Med Hyg. 2020;103(2):876. https://doi.org/10.4269/ajtmh.19-0698.

69. Ayers JB, Xie X, Coatsworth H, Stephenson CJ, Waits CM, Shi PY, et al. Infection kinetics and transmissibility of a reanimated dengue virus serotype 4 identified originally in wild Aedes aegypti from Florida. Front Microbiol. 2021;12:734903. https://doi.org/10.3389/fmicb.2021.734903.

70. Lai Z, Zhou T, Zhou J, Liu S, Xu Y, Gu J, et al. VT of Zika virus in Aedes albopictus. PLoS Negl Trop Dis. 2020;14(10):e0008776. https://doi.org/10.1371/journal.pntd.0008776.

71. Nuñez AI, Talavera S, Birnberg L, Rivas R, Pujol N, Verdún M, et al. Evidence of Zika virus horizontal and vertical transmission in Aedes albopictus from Spain but not infectious virus in saliva of the progeny. Emerg Microbes Infect. 2020;9(1):2236-44. https://doi.org/10.1080/22221751.2020.1830718.

72. Yang SC, Lee HI, Kim H, Lee WG. Transmission ability of Zika virus with artificially infected Aedes albopictus in Korea. Entomol Res. 2021;51(8):413-20. https://doi.org/10.1111/1748-5967.12539.

73. Honório NA, Wiggins K, Eastmond B, Câmara DCP, Alto BW. Experimental vertical transmission of Chikungunya virus by Brazilian and Florida Aedes albopictus populations. Viruses. 2019;11(4):353. https://doi.org/10.3390/v11040353.

74. Anderson JF, Main AJ, Ferrandino FJ. Horizontal and vertical transmission of virus by Aedes vexans (Diptera: Culicidae). J Med Entomol. 2020;57(5):1614-8. https://doi.org/10.1093/jme/tjaa049.

75. Anderson JF, Main AJ, Delroux K, Fikrig E. Extrinsic incubation periods for horizontal and vertical transmission of West Nile virus by Culex pipiens pipiens (Diptera: Culicidae). J Med Entomol. 2014;45(3):445-51.https:// DOI: 10.1603/0022-2585(2008)45[445:eipfha]2.0.co;2

76. Chompoosri J, Thavara U, Tawatsin A, Boonserm R, Phumee A, Sangkitporn S, et al. VT of Indian Ocean Lineage of Chikungunya virus in Aedes aegypti and Aedes albopictus mosquitoes. Parasites Vectors. 2016;9:1-13. https://doi.org/10.1186/s13071-016-1505-6.

77. Hernández-Triana LM, Barrero E, Delacour-Estrella S, et al. Evidence for infection but not transmission of Zika virus by Aedes albopictus (Diptera: Culicidae) from Spain. Parasites Vectors. 2019;12:204. https://doi.org/10.1186/s13071-019-3467-y.

78. Zimler RA, Alto BW. Vertical transmission of Zika Virus by Florida Aedes aegypti and Ae. albopictus. Insects. 2023;14(3):289. https://doi.org/10.3390/insects14030289.

79. Fortuna C, Remoli ME, Di Luca M, et al. Experimental studies on comparison of the vector competence of four Italian Culex pipiens populations for West Nile virus. Parasites Vectors. 2015;8:463. https://doi.org/10.1186/s13071-015-1067-z.

80. Eastwood G, Cunningham AA, Kramer LD, Goodman SJ. The vector ecology of introduced Culex quinquefasciatus populations, and implications for future risk of West Nile virus emergence in the Galápagos archipelago. Med Vet Entomol. 2019;33(1):44-55. https://doi.org/10.1111/mve.12329.

81. Buckner EA, Alto BW, Lounibos LP. Vertical transmission of Key West dengue-1 virus by Aedes aegypti and Aedes albopictus (Diptera: Culicidae) mosquitoes from Florida. J Med Entomol. 2013;50(6):1291-7. https://doi.org/10.1603/me13047.

82. Phumee A, Chompoosri J, Intayot P, Boonserm R, Boonyasuppayakorn S, Buathong R, et al. Vertical transmission of Zika virus in Culex quinquefasciatus Say and Aedes aegypti (L.) mosquitoes. Sci Rep. 2019;9(1):5257. https://doi.org/10.1038/s41598-019-41727-8.

83. Nag DK, Payne AF, Dieme C, Ciota AT, Kramer LD. Zika virus infects Aedes aegypti ovaries. Virology. 2021;561:58-64. doi: 10.1016/j.virol.2021.06.002.

84. Diallo, M., Thonnon, J. and Fontenille, D., 2000. Vertical transmission of the yellow fever virus by Aedes aegypti (Diptera, Culicidae): dynamics of infection in F1 adult progeny of orally infected females. The American journal of tropical medicine and hygiene, 62(1), pp.151-156.

85. Thangamani S, Huang J, Hart CE, Guzman H, Tesh RB. Vertical transmission of Zika virus in Aedes aegypti mosquitoes. Am J Trop Med Hyg. 2016;95(5):1169. doi: 10.4269/ajtmh.16-0448.

86. Khin, M.M. and Than, K.A., 1983. Transovarial transmission of dengue 2 virus by Aedes aegypti in nature. The American journal of tropical medicine and hygiene, 32(3), pp.590-594.

87. Hull, B., Tikasingh, E., de Souza, M. and Martinez, R., 1984. Natural transovarial transmission of dengue 4 virus in Aedes aegypti in Trinidad. The American journal of tropical medicine and hygiene, 33(6), pp.1248-1250.

88. Watts, D.M., Harrison, B.A., Pantuwatana, S., Klein, T.A. and Burke, D.S., 1985. Failure to detect natural transovarial transmission of dengue viruses by Aedes aegypti and Aedes albopictus (Diptera: Culicidae). Journal of medical entomology, 22(3), pp.261-265.

89. Dhileepan, K., Azuolas, J.K. and Gibson, C.A., 1996. Evidence of vertical transmission of Ross River and Sindbis viruses (Togaviridae: Alphavirus) by mosquitoes (Diptera: Culicidae) in southeastern Australia. Journal of medical entomology, 33(1), pp.180-182.

90. Fontenille, D., Diallo, M., Mondo, M., Ndiaye, M. and Thonnon, J., 1997. First evidence of natural vertical transmission of yellow fever virus in Aedes aegypti, its epidemic vector. Transactions of the Royal Society of Tropical Medicine and Hygiene, 91(5), pp.533-535.

91. LEAKE and FALCONAR, 1998. Determination of dengue virus serotypes in individual Aedes aegypti mosquitoes in Colombia. Medical and veterinary entomology, 12(3), pp.284-288.

92. Phillips, R.A. and Christensen, K., 2006. Field-caught Culex erythrothorax larvae found naturally infected with West Nile virus in Grand County, Utah. Journal of the American Mosquito Control Association, 22(3), pp.561-562.

93. Joshi, V., Sharma, R.C., Sharma, Y., Adha, S., Sharma, K., Singh, H., Purohit, A. and Singhi, M., 2006. Importance of socioeconomic status and tree holes in distribution of Aedes mosquitoes (Diptera: Culicidae) in Jodhpur, Rajasthan, India. Journal of medical entomology, 43(2), pp.330-336.

94. Thenmozhi, V., Hiriyan, J.G., Tewari, S.C., Samuel, P.P., Paramasivan, R., Rajendran, R., Mani, T.R. and Tyagi, B.K., 2007. Natural vertical transmission of dengue virus in Aedes albopictus (Diptera: Culicidae) in Kerala, a southern Indian state. Japanese journal of infectious diseases, 60(5), pp.245-249.

95. Arunachalam, N., Tewari, S.C., Thenmozhi, V., Rajendran, R., Paramasivan, R., Manavalan, R., Ayanar, K. and Tyagi, B.K., 2008. Natural vertical transmission of dengue viruses by Aedes aegypti in Chennai, Tamil Nadu, India. Indian Journal of Medical Research, 127(4), pp.395-407.

96. McAbee, R.D., Green, E.N., Holeman, J., Christiansen, J., Frye, N., Dealey, K., Mulligan, F.S., Brault, A.C. and Cornel, A.J., 2008. Identification of Culex pipiens complex mosquitoes in a hybrid zone of West Nile virus transmission in Fresno County, California. The American journal of tropical medicine and hygiene, 78(2), pp.303-310.

97. Zeidler, J.D., Acosta, P.O.A., Barrêto, P.P. and Cordeiro, J.D.S., 2008. Dengue virus in Aedes aegypti larvae and infestation dynamics in Roraima, Brazil. Revista de saúde pública, 42, pp.986-991.

98. Cecílio, A.B., Campanelli, E.S., Souza, K.P.R., Figueiredo, L.B. and Resende, M., 2009. Natural vertical transmission by Stegomyia albopicta as dengue vector in Brazil. Brazilian Journal of Biology, 69, pp.123-127.

99. Widiarti, Boewono, D.T. and Widyastuti, U., 2009. THE DETECTION OF DENGUE ANTIGEN IN PROGENY DENGUE HEMORRHAGIC FEVER MOSQUITO VECTORS USING IMUNOHISTOCHEMICAL ASSAY. BULETIN PENELITIAN KESEHATAN, 37(3), pp.126-136.

100. Unlu, I., Mackay, A.J., Roy, A., Yates, M.M. and Foil, L.D., 2010. Evidence of vertical transmission of West Nile virus in field-collected mosquitoes. Journal of Vector Ecology, 35(1), pp.95-99.

101. Flores, F.S., Diaz, L.A., Batallán, G.P., Almirón, W.R. and Contigiani, M.S., 2010. vertical transmission of St. Louis encephalitis virus in Culex quinquefasciatus (Diptera: Culicidae) in Córdoba, Argentina. Vector-Borne and Zoonotic Diseases, 10(10), pp.999-1002.

102.Hartanti, M.D., Suryani, S. and Tirtadjaja, I.A., 2010. Dengue virus transovarial transmission by Aedes aegypti. Universa Med icina, 29(2), pp.65-70.

103.Thongrungkiat, S., Wasinpiyamongkol, L., Maneekan, P., Prummongkol, S. and Samung, Y., 2012. Natural transovarial dengue viru s infection rate in both sexes of dark and pale forms of Aedes aegypti from an urban area of Bangkok, Thailand. Southeast Asian Journal of Tropical Medicine & Public Health, 43(5), pp.1146-1152.

104.Rohani, A., Azahary, A.A., Malinda, M., Zurainee, M.N., Rozilawati, H., Najdah, W.W. and Lee, H.L., 2014. Eco -virological survey of Aedes mosquito larvae in selected dengue outbreak areas in Malaysia. Journal of vector borne diseases, 51(4), pp.327 -332.

105.Afzal, S., Maqbool, A., Zaman, S., Idrees, M., Anjum, A.A., Shah, S.S., Fatima, Z., Amin, I. and Latif, M., 2015. Evidence of transmission of dengue virus by different developmental stages of mosquitoes, Aedes aegypti and Aedes albopictus. Pakistan J. Zool , 47(3), pp.887-890.

106.Edillo, F.E., Sarcos, J.R. and Sayson, S.L., 2015. Natural vertical transmission of dengue viruses in Aedes aegypti in select ed sites in Cebu City, Philippines. Journal of Vector Ecology, 40(2), pp.282-291.

107.Dutta, P., Khan, S.A., Chetry, S., Dev, V., Sarmah, C.K. and Mahanta, J., 2015. First evidence of dengue virus infection in wild caught mosquitoes during an outbreak in Assam, Northeast India. Journal of vector borne diseases, 52(4), pp.293 -298.

108.Vikram, K., Nagpal, B.N., Pande, V., Srivastava, A., Saxena, R., Singh, H., Gupta, S.K., Tuli, N.R., Yadav, N.K., Olivier, T. and Richard, P., 2015. Detection of dengue virus in individual Aedes aegypti mosquitoes in Delhi, India. Journal of vector borne d iseases, 52(2), pp.129-133.

109.Moura, A.J.F.D., de Melo Santos, M.A.V., Oliveira, C.M.F., Guedes, D.R.D., de Carvalho -Leandro, D., da Cruz Brito, M.L., Rocha, H.D.R., Gómez, L.F. and Ayres, C.F.J., 2015. Vector competence of the Aedes aegypti population from Santiago Island, Cape Verde, to different serotypes of dengue virus. Parasites & vectors, 8, pp.1-9.

110.Westby, K.M., Fritzen, C., Paulsen, D., Poindexter, S. and Moncayo, A.C., 2015. La Crosse encephalitis virus infection in fie ld-collected Aedes albopictus, Aedes japonicus, and Aedes triseriatus in Tennessee. *Journal of the American Mosquito Control Association*, *31*(3), pp.233-241

111.Tingström, O., Wesula Lwande, O., Näslund, J., Spyckerelle, I., Engdahl, C., Von Schoenberg, P., Ahlm, C., Evander, M. and Bu cht, G., 2016. Detection of Sindbis and Inkoo virus RNA in genetically typed mosquito larvae sampled in Northern Sweden. Vector-Borne and Zoonotic Diseases, 16(7), pp.461 -467.

112.Angel, A., Angel, B. and Joshi, V., 2016. Rare occurrence of natural transovarial transmission of dengue virus and elimination of infected foci as a possible intervention method. Acta tropica, 155, pp.20 -24.

113.Khan, J., Khan, I., Ijaz, A., Iqbal, A. and Salman, M., 2017. The role of vertical transmission of dengue virus among field -captured Aedes aegypti and Aedes albopictus mosquitoes in Peshawar, Khyber Pakhtunkhwa, Pakistan. Pakistan Journal of Zoology, 49(3).

114. Smartt, C.T., Stenn, T.M., Chen, T.Y., Teixeira, M.G., Queiroz, E.P., Souza Dos Santos, L., Queiroz, G.A., Ribeiro Souza, K., Kalabric Silva, L., Shin, D. and Tabachnick, W.J., 2017. Evidence of Zika virus RNA fragments in Aedes albopictus (Diptera: Culicidae) field-collected eggs from Camaçari, Bahia, Brazil. Journal of Medical Entomology, 54(4), pp.1085-1087.

115. Costa, C.F.D., Silva, A.V.D., Nascimento, V.A.D., Souza, V.C.D., Monteiro, D.C.D.S., Terrazas, W.C.M., Dos Passos, R.A., Nascimento, S., Lima, J.B.P. and Naveca, F.G., 2018. Evidence of vertical transmission of Zika virus in field-collected eggs of Aedes aegypti in the Brazilian Amazon. PLoS Neglected Tropical Diseases, 12(7), p.e0006594.

116. Ajamma, Y.U., Onchuru, T.O., Ouso, D.O., Omondi, D., Masiga, D.K. and Villinger, J., 2018. Vertical transmission of naturally occurring Bunyamwera and insect-specific flavivirus infections in mosquitoes from islands and mainland shores of Lakes Victoria and Baringo in Kenya. PLoS Neglected Tropical Diseases, 12(11), p.e0006949.

117. Ponce-García, G., Flores-Suarez, A.E., Villanueva-Segura, K., Lopez-Rodriguez, M., Dzul, F., Lopez-Monroy, B. and Rodriguez-Sanchez, I., 2018. Report of Chikungunya virus in wild populations of Aedes aegypti in Guerrero State, Mexico. Journal of the American Mosquito Control Association, 34(2), pp.147-150.

118. Kobayashi, D., Murota, K., Fujita, R., Itokawa, K., Kotaki, A., Moi, M.L., Ejiri, H., Maekawa, Y., Ogawa, K., Tsuda, Y. and Sasaki, T., 2018. Dengue virus infection in Aedes albopictus during the 2014 autochthonous dengue outbreak in Tokyo Metropolis, Japan. The American journal of tropical medicine and hygiene, 98(5), p.1460.

119. Garcia-Rejon, J.E., Ulloa-Garcia, A., Cigarroa-Toledo, N., Pech-May, A., Machain-Williams, C., Cetina-Trejo, R.C., Talavera-Aguilar, L.G., Torres-Chable, O.M., Navarro, J.C. and Baak-Baak, C.M., 2018. Study of Aedes aegypti population with emphasis on the gonotrophic cycle length and identification of arboviruses: implications for vector management in cemeteries. Revista do Instituto de Medicina Tropical de São Paulo, 60, p.e44.

120. Izquierdo-Suzán, M., Zárate, S., Torres-Flores, J., Correa-Morales, F., González-Acosta, C., Sevilla-Reyes, E.E., Lira, R., Alcaraz-Estrada, S.L. and Yocupicio-Monroy, M., 2019. Natural vertical transmission of Zika virus in larval Aedes aegypti populations, Morelos, Mexico. Emerging Infectious Diseases, 25(8), p.1477.

121. Satoto, T.B., Wati, N.A.P., Purwaningsih, W., Josef, H.K., Purwono, P., Rumbiwati, R., Hermanto, H. and Frutos, R., 2019. Occurrence of natural vertical transmission of "Zika like Virus" in Aedes aegypti mosquito in Jambi City. Kesmas: Jurnal Kesehatan Masyarakat Nasional (National Public Health Journal), 13(4), pp.189-194.

122. Rua-Uribe, G.L., Giraldo-Jaramillo, T.M., Triana-Chavez, O., Rojo, R., Henao, E. and Perez-Perez, J., 2020. Vertical transmission of dengue virus in Aedes spp.(Diptera: Culicidae) in Medellín, Colombia. Revista Colombiana de Entomología, 46(1).

123. Diouf, B., Gaye, A., Diagne, C.T., Diallo, M. and Diallo, D., 2020. Zika virus in southeastern Senegal: survival of the vectors and the virus during the dry season. BMC Infectious Diseases, 20, pp.1-9.

124. Harvie, S., Nor Aliza, A.R., Lela, S. and Razitasham, S., 2020. Detection of dengue virus serotype 2 (DENV-2) in population of Aedes mosquitoes from Sibu and Miri divisions of Sarawak using reverse transcription polymerase chain reaction (RT-PCR) and semi-nested PCR.

125.Wijesinghe, C., Gunatilake, J., Kusumawathie, P.H.D., Sirisena, P.D.N.N., Daulagala, S.W.P.L., Iqbal, B.N. and Noordeen, F., 2021. Circulating dengue virus serotypes and vertical transmission in Aedes larvae during outbreak and inter-outbreak seasons in a high dengue risk area of Sri Lanka. Parasites & vectors, 14(1), p.614.

126.Sudarmaja, I.M., Swastika, I.K., Diarthini, L.P.E., Prasetya, I.P.D. and Wirawan, I.M.A., 2022. Dengue virus transovarial transmission detection in Aedes aegypti from dengue hemorrhagic fever patients' residences in Denpasar, Bali. Veterinary world, 15(4), p.1149.

127.Kurnia, N., Kaitana, Y., Salaki, C.L., Mandey, L.C., Tuda, J.S.B. and Tallei, T.E., 2022. Study of dengue virus transovarial transmission in aedes spp. in ternate city using streptavidin-biotin-peroxidase complex immunohistochemistry. Infectious Disease Reports, 14(5), pp.765-771.

128.Silva, D.M.F.D., Curcio, J.S.D., Silva, L.D.C., Sousa, F.B.D., Anunciação, C.E., Furlaneto, S.M.S.I., Silva, V.P.S.M., Garcia-Zapata, M.T.A. and Silveira-Lacerda, E.D.P., 2024. Detection of arboviruses in Aedes aegypti through transovarian analysis: A study in Goiânia, Goiás. Revista da Sociedade Brasileira de Medicina Tropical, 57, pp.e00400-2023.

129.Ferreira-de-Lima, V.H., Andrade, P.D.S., Thomazelli, L.M., Marrelli, M.T., Urbinatti, P.R., Almeida, R.M.M.D.S. and Lima-Camara, T.N., 2020. Silent circulation of dengue virus in Aedes albopictus (Diptera: Culicidae) resulting from natural vertical transmission. Scientific reports, 10(1), p.3855.

130.Chaves BA, Junior ABV, Silveira KRD, Paz ADC, Vaz EBDC, Araujo RGP, et al. Vertical transmission of Zika virus (Flaviviridae, Flavivirus) in Amazonian Aedes aegypti (Diptera: Culicidae) delays egg hatching and larval development of progeny. J Med Entomol. 2019;56(6):1739-44. https://doi.org/10.1093/jme/tjz110

131.Jain J, Kushwah RBS, Singh SS, Sharma A, Adak T, Singh OP, et al. Evidence for natural vertical transmission of Chikungunya viruses in field populations of Aedes aegypti in Delhi and Haryana states in India—a preliminary report. Acta Trop. 2016;162:46-55. doi: 10.1016/j.actatropica.2016.06.004.

132.Thongrungkiat S, Maneekan P, Wasinpiyamongkol L, Prummongkol S. Prospective field study of transovarial dengue-virus transmission by two different forms of Aedes aegypti in an urban area of Bangkok, Thailand. J Vector Ecol. 2011;36(1):147-52. doi: 10.1111/j.1948-7134.2011.00151.x.

133.Moraes A, Cortelli FC, Miranda TB, Aquino DR, Cortelli JR, Guimarães MIA, et al. Transovarial transmission of dengue 1 virus in Aedes aegypti larvae: real-time PCR analysis in a Brazilian city with high mosquito population density. Can J Microbiol. 2018;64(6):393-400. doi: 10.1139/cjm-2017-0614.

134.Günther J, Martínez-Muñoz JP, Pérez-Ishiwara DG, Salas-Benito J. Evidence of vertical transmission of dengue virus in two endemic localities in the state of Oaxaca, Mexico. Intervirology. 2007;50(5):347-52. doi: 10.1159/000107272.

135.Heath CJ, Grossi-Soyster EN, Ndenga BA, Mutuku FM, Sahoo MK, Ngugi HN, et al. Evidence of transovarian transmission of Chikungunya and dengue viruses in field-caught mosquitoes in Kenya. PLoS Negl Trop Dis. 2020;14(6):e0008362. doi: 10.1371/journal.pntd.0008362.

136.Sanchez-Rodríguez OS, Sanchez-Casas RM, Laguna-Aguilar M, Alvarado-Moreno MS, Zarate-Nahon EA, Ramirez-Jimenez R, et al. Natural transmission of dengue virus by Aedes albopictus at Monterrey, Northeastern Mexico. Southwest Entomol. 2014;39(3):459-68. doi: 10.3958/059.039.0307.

137. Farraudière L, Sonor F, Crico S, Étienne M, Mousson L, Hamel R, et al. First detection of dengue and chikungunya viruses in natural populations of Aedes aegypti in Martinique during the 2013–2015 concomitant outbreak. Rev Panam Salud Publica. 2017;41:e63. doi: 10.26633/RPSP.2017.63.

138. Sanchez-Casas RM, Gaitan-Burns A, Diaz-Gonzalez EE, Grajales JS, Dector MA, Fernandez-Salas I. Evidence of DENV-2 vertical transmission in larval Aedes aegypti populations at Cancun, Quintana Roo, Mexico. Southwest Entomol. 2016;41(2):389-98. doi: 10.3958/059.041.0204.

139. Costa CFD, Silva AVD, Nascimento VAD, Souza VCD, Monteiro DCDS, Terrazas WCM, et al. Evidence of VT of Zika virus in field-collected eggs of Aedes aegypti in the Brazilian Amazon. PLoS Negl Trop Dis. 2018;12(7):e0006594. doi: 10.1371/journal.pntd.0006594.

140. Torres-Avendaño JI, Apodaca-Medina AI, Castillo-Ureta H, Rendón-Maldonado JG, Torres-Montoya EH, Cota-Medina A, et al. Natural vertical transmission of dengue virus serotype 4 in Aedes aegypti larvae from urban areas in Sinaloa, Mexico. Vector Borne Zoonotic Dis. 2021;21(6):478-481. https://doi.org/10.1089/vbz.2020.2748

141. Maia LMS, Bezerra MCF, Costa MCS, Souza EM, Oliveira MEB, Ribeiro ALM, et al. Natural vertical infection by dengue virus serotype 4, Zika virus and Mayaro virus in Aedes aegypti and Aedes albopictus. Med Vet Entomol. 2019;33(3):437-442. https://doi.org/10.1111/mve.12369

142. Vilela AP, Figueiredo LB, dos Santos JR, Eiras AE, Bonjardim CA, Ferreira PC, Kroon EG. Dengue virus 3 genotype I in Aedes aegypti mosquitoes and eggs, Brazil, 2005–2006. Emerg Infect Dis. 2010;16(6):989. https://doi.org/10.3201/eid1606.091000

143. de Figueiredo ML, de C Gomes A, Amarilla AA, de S Leandro A, de S Orrico A, de Araujo RF, et al. Mosquitoes infected with dengue viruses in Brazil. Virol J. 2010;7:1-5. https://doi.org/10.1186/1743-422X-7-152

144. Kolodziejek J, Seidel B, Jungbauer C, Dimmel K, Kolodziejek M, Rudolf I, et al. West Nile virus positive blood donation and subsequent entomological investigation, Austria, 2014. PLoS One. 2015;10(5).https:// doi.org/10.1371/journal.pone.0126381

145. Zeidler JD, Acosta POA, Barrêto PP, Cordeiro JDS. Dengue virus in Aedes aegypti larvae and infestation dynamics in Roraima, Brazil. Rev Saude Publica. 2008;42:986-991. https://doi.org/10.1590/S0034-89102008005000055

146. Le Goff G, Revollo J, Guerra M, Cruz M, Simon ZB, Roca Y, et al. Natural vertical transmission of dengue viruses by Aedes aegypti in Bolivia. Parasite. 2011;18(3):277. https://doi.org/10.1051/parasite/2011183277

147. Fechter-Leggett E, Nelms BM, Barker CM, Reisen WK. West Nile virus cluster analysis and vertical transmission in Culex pipiens complex mosquitoes in Sacramento and Yolo Counties, California, 2011. J Vector Ecol. 2012;37(2):442-449. https://doi.org/10.1111/j.1948-7134.2012.00248.x

148. da Costa CF, Dos Passos RA, Lima JBP, Roque RA, de Souza Sampaio V, Campolina TB, et al. Transovarial transmission of dengue in Aedes aegypti in the Amazon basin: a local model of xenomonitoring. Parasit Vectors. 2017;10:1-9. https://doi.org/10.1186/s13071-017-2194-5

149. Martins VEP, Alencar CH, Kamimura MT, de Carvalho Araujo FM, De Simone SG, Dutra RF, Guedes MIF. Occurrence of natural vertical transmission of dengue-2 and dengue-3 viruses in Aedes aegypti and Aedes albopictus in Fortaleza, Ceará, Brazil. PLoS One. 2012;7(7):e41386. https://doi.org/10.1371/journal.pone.0041386

150. Pinheiro V, Tadei WP, Barros PM, Vasconcelos PF, Cruz ACR. Detection of dengue virus serotype 3 by reverse transcription-polymerase chain reaction in Aedes aegypti (Diptera, Culicidae) captured in Manaus, Amazonas. Mem Inst Oswaldo Cruz. 2005;100:833-839. https://doi.org/10.1590/S0074-02762005000800003

151. Alencar J, Ferreira de Mello C, Brisola Marcondes C, Érico Guimarães A, Toma HK, Queiroz Bastos A, et al. Natural infection and vertical transmission of Zika virus in sylvatic mosquitoes Aedes albopictus and Haemagogus leucocelaenus from Rio de Janeiro, Brazil. Trop Med Infect Dis. 2021;6(2):99. https://doi.org/10.3390/tropicalmed6020099

152. Cruz LC, Serra OP, Leal-Santos FA, Ribeiro AL, Slhessarenko RD, Santos MA. Natural transovarial transmission of dengue virus 4 in Aedes aegypti from Cuiabá, Mato Grosso, Brazil. Rev Soc Bras Med Trop. 2015;48(1):18-25. https://doi.org/10.1590/0037-8682-0264-2014

153. Ruiz-López F, González-Mazo A, Vélez-Mira A, Gómez GF, Zuleta L, Uribe S, Vélez-Bernal ID. Presence of Aedes aegypti and its natural infection with dengue virus at unrecorded heights in Colombia. Biomedica. 2016;36(2):303-309. https://doi.org/10.7705/biomedica.v36i2.3301

154. Teixeira AF, de Brito BB, Correia TML, Viana AIS, Carvalho JC, da Silva FAF, et al. Simultaneous circulation of Zika, Dengue, and Chikungunya viruses and their vertical co-transmission among Aedes aegypti. Acta Trop. 2021;215:105819. https://doi.org/10.1016/j.actatropica.2020.105819

155. Gutiérrez-Bugallo G, Rodríguez-Roche R, Díaz G, Perez M, Mendizábal ME, Peraza I, et al. Spatio-temporal distribution of vertically transmitted dengue viruses by Aedes aegypti from Arroyo Naranjo, Havana, Cuba. Trop Med Int Health. 2018;23(12):1342-1349. https://doi.org/10.1111/tmi.13162

156. Cecílio SG, Júnior WFS, Tótola AH, de Brito Magalhães CL, Ferreira JMS, de Magalhães JC. Dengue virus detection in Aedes aegypti larvae from southeastern Brazil. J Vector Ecol. 2015;40(1):71-74. https://doi.org/10.1111/jvec.12134

157. Cecílio AB, Campanelli ES, Souza KPR, Figueiredo LB, Resende MC. Natural vertical transmission of Stegomyia albopicta as a vector of dengue in Brazil. Braz J Biol. 2009;69:123-127. https://doi.org/10.1590/S1519-69842009000100015

158. Granger Neto HP, Rocha CV, Correia TML, Silva NMPD, Chaves BA, Secundino NFC, et al. Natural vertical co-transmission of Dengue virus and Chikungunya virus from Aedes aegypti in Brumado, Bahia, Brazil. Rev Soc Bras Med Trop. 2022;55:e0427-2021. https://doi.org/10.1590/0037-8682-0427-2021

159. Pessanha JEM, Caiaffa WT, Cecilio AB, Iani FCDM, Araujo SC, Nascimento JC, et al. Cocirculation of two dengue virus serotypes in individual and pooled samples of Aedes aegypti and Aedes albopictus larvae. Rev Soc Bras Med Trop. 2011;44:103-105. https://doi.org/10.1590/S0037-86822011000100023

160. Gutiérrez-Bugallo G, Rodríguez-Roche R, Díaz G, Vázquez AA, Alvarez M, Rodríguez M, et al. First record of natural vertical transmission of dengue virus in Aedes aegypti from Cuba. Acta Trop. 2017;174:146-148. https://doi.org/10.1016/j.actatropica.2017.07.012

161. Piedra LA, Pupo M, Rodríguez MM, Fraga J, Guzmán MG, Kourí V. Development of loop-mediated isothermal amplification assay for rapid detection of dengue virus in Aedes aegypti (Diptera: Culicidae) larvae from cuba. Virology & Retrovirology Journal. 2019;2(2):122.

162. Vilela AP, Figueiredo LB, dos Santos JR, Eiras AE, Bonjardim CA, Ferreira PC, et al. Dengue virus 3 genotype I in Aedes aegypti mosquitoes and eggs, Brazil, 2005–2006. Emerg Infect Dis. 2010;16(6):989. https://doi.org/10.3201/eid1606.091000

163. Piedra LA, Martinez LC, Ruiz A, Vazquez JR, Guzman MG, Rey J, et al. First record of natural transovarial transmission of Dengue virus in Aedes albopictus from Cuba. Am J Trop Med Hyg. 2022. https://doi.org/10.4269/ajtmh.21-0710

164. Martínez NE, Dzul-Manzanilla F, Gutiérrez-Castro C, Ibarra-López J, Bibiano-Marín W, López-Damián L, et al. Natural vertical transmission of dengue-1 virus in Aedes aegypti populations in Acapulco, Mexico. J Am Mosq Control Assoc. 2014;30(2):143-146. https://doi.org/10.2987/14-6402.1

165. Danis-Lozano R, Díaz-González EE, Malo-García IR, Rodríguez MH, Ramos-Castañeda J, Juárez-Palma L, et al. Vertical transmission of dengue virus in Aedes aegypti and its role in the epidemiological persistence of dengue in Central and Southern Mexico. Trop Med Int Health. 2019;24(11):1311-1319. https://doi.org/10.1111/tmi.13306

166. Rudolf, I., Betášová, L., Blažejová, H., Venclíková, K., Straková, P., Šebesta, O., Mendel, J., Bakonyi, T., Schaffner, F., Nowotny, N. and Hubálek, Z., 2017. West Nile virus in overwintering mosquitoes, central Europe. Parasites & vectors, 10, pp.1-4. https:// doi: 10.1186/s13071-017-2399-7

167. Fouque, F., Garinci, R. and Gaborit, P., 2004. Epidemiological and entomological surveillance of the co-circulation of DEN-1, DEN-2 and DEN-4 viruses in French Guiana. Tropical Medicine & International Health, 9(1), pp.41-46.

168. Guedes, D.R.D., Cordeiro, M.T., Melo-Santos, M.A.V., Magalhaes, T., Marques, E., Regis, L., Furtado, A.F. and Ayres, C.F.J., 2010. Patient-based dengue virus surveillance in Aedes aegypti from Recife, Brazil. Journal of vector borne diseases, 47(2), p.67.

169. Chompoosri, J., Thavara, U., Tawatsin, A., Anantapreecha, S. and Siriyasatien, P., 2012. Seasonal monitoring of dengue infection in Aedes aegypti and serological feature of patients with suspected dengue in 4 central provinces of Thailand. The Thai Journal of Veterinary Medicine, 42(2), pp.185-193.

170. Lucia Velandia-Romero, M., Alberto Olano, V., Coronel-Ruiz, C., Cabezas, L., Angelica Calderon-Pelaez, M., Eduardo Castellanos, J. and Ines Matiz, M., 2017. Dengue virus detection in larvae and pupae of Aedes aegypti collected in a rural area of Anapoima, Cundinamarca, Colombia. BIOMEDICA, 37.

171. Satoto, T.B.T., Listyantanto, A., Agustjahjani, S.D., Josef, H.K. and Widartono, B.S., 2018. Vertical transmission of dengue virus in the Yogyakarta airport area. Environmental health and preventive medicine, 23, pp.1-7.

172. Niyas, K.P., Abraham, R., Unnikrishnan, R.N., Mathew, T., Nair, S., Manakkadan, A., Issac, A. and Sreekumar, E., 2010. Molecular characterization of Chikungunya virus isolates from clinical samples and adult Aedes albopictus mosquitoes emerged from larvae from Kerala, South India. Virology Journal, 7, pp.1-8.

173. Dahl E, Öborn L, Sjöberg V, Lundkvist Å, Hesson JC. Vertical transmission of Sindbis virus in Culex mosquitoes. Viruses. 2022;14(9):1915. https://doi.org/10.3390/v14091915

174. Nelms BM, Macedo PA, Kothera L, Savage HM, Reisen WK. Overwintering biology of Culex (Diptera: Culicidae) mosquitoes in the Sacramento valley of California. J Med Entomol. 2013;50(4):773-790. https://doi.org/10.1603/me12280

175. Reisen WK, Fang Y, Lothrop HD, Martinez VM, Wilson J, O'Connor P, et al. Overwintering of West Nile virus in southern California. J Med Entomol. 2014;43(2):344-355. https://doi.org/10.1093/jmedent/43.2.344
