# Supplementary material for: Correction: Significance of vertical transmission of arboviruses in mosquito-borne disease epidemiology
Source: Parasit Vectors. 2025 Jun 18;18:226. doi: 10.1186/s13071-025-06864-2 (PMC12175438; doi:10.1186/s13071-025-06864-2)
Supplement: Supplementary file 1 — Supplementary material 1. [file 13071_2025_6864_MOESM1_ESM.pdf]

Parasites & Vectors

REVIEW Open Access

Significance of vertical transmission of arboviruses in mosquito-borne disease epidemiology

Oliver Chinonso Mbaoma[1*], Stephanie Margarete Thomas[1,2] and Carl Beierkuhnlein[1,2]

Abstract

Mosquito-borne diseases (MBDs) are increasingly prevalent due to the resultant impact of global change with significant health and economic impacts worldwide. Dengue virus (DENV), chikungunya virus (CHIKV), Zika virus (ZIKV), yellow fever virus (YFV), Japanese encephalitis (JEV), and West Nile virus (WNV) transmitted by *Aedes* and *Culex* species have been identified as arboviruses of public health interest. The vertical transmission (VT) refers to the process where infected mosquitoes transmit viruses to their offspring; this has been often overlooked in MBD epidemiology. We conducted a systematic review to evaluate the role of VT in the occurrence, prevalence, and spread of MBDs, focusing on study types, mosquito species, and virus genera. In total, 73 studies from 2005 to 2024 relating to VT in the mosquito population were reviewed. Findings revealed the occurrence of VT across multiple mosquito species in natural and experimental settings, with significant variation in VT rates depending on vector species, virus genus, and study location. *Aedes aegypti*, *Aedes albopictus*, *Aedes vexans*, *Culex pipiens*, *Culex tarsalis*, and *Culex quinquefasciatus* were identified as mosquito species that support VT, while pathogens identified to be transmitted vertically were DENV, ZIKV, WNV, CHIKV, YFV, Sindbis virus (SINV), Ross River virus (RRV), and Mayaro virus (MAYV). VT rates were reported as minimum, and infection rate (MIR) varied across species, study type and location. Also, a high VT rate may precede a mosquito-borne disease outbreak. These findings indicate that VT, though often overlooked, contributes to the dynamics of MBD transmission and could influence disease outbreaks and endemism, especially under changing climatic conditions, highlighting the need for incorporating VT in mathematical models, experimental studies, and control strategies to understand dynamics of MBDs, given its potential role in sustaining arbovirus transmission and influencing outbreak dynamics.

Keywords Mosquito-borne diseases, Vector-borne diseases, One health, Vertical transmission, Global change

*Correspondence:
Oliver Chinonso Mbaoma
Oliver.mbaoma@uni-bayreuth.de
[1] Department of Biogeography, University of Bayreuth, Bayreuth, Germany
[2] Center of Ecology and Environmental Research, BayCEER, University of Bayreuth, Bayreuth, Germany

Background

Mosquito-borne diseases, mosquitoes, and pathogens of interest

The occurrence, spread, and prevalence of mosquito-borne diseases (MBDs) has been increasing across the globe. This increase has been attributed to changes in climatic conditions and globalization [1]. MBDs have been implicated as a major cause of mortality and morbidity with significant economic and health impacts [2]. They are transmitted to humans and animal hosts by competent vector mosquitoes. *Aedes* and *Culex* mosquitoes have

© The Author(s) 2025. **Open Access** This article is licensed under a Creative Commons Attribution 4.0 International License, which permits use, sharing, adaptation, distribution and reproduction in any medium or format, as long as you give appropriate credit to the original author(s) and the source, provide a link to the Creative Commons licence, and indicate if changes were made. The images or other third party material in this article are included in the article's Creative Commons licence, unless indicated otherwise in a credit line to the material. If material is not included in the article's Creative Commons licence and your intended use is not permitted by statutory regulation or exceeds the permitted use, you will need to obtain permission directly from the copyright holder. To view a copy of this licence, visit http://creativecommons.org/licenses/by/4.0/. The Creative Commons Public Domain Dedication waiver (http://creativecommons.org/publicdomain/zero/1.0/) applies to the data made available in this article, unless otherwise stated in a credit line to the data.

been identified as competent mosquitoes for several arboviruses of public health interest. About 73% of emerging and re-emerging pathogens are arboviruses transmitted by mosquitoes, some of which have been identified as very prevalent due to the rate at which they break out and spread to new locations, and the difficulty encountered in attempts to control them [3, 4]. Regardless, of over 500 arboviruses recognized, Dengue virus (DENV), chikungunya virus (CHIKV), Zika virus (ZIKV), yellow fever virus (YFV), Japanese encephalitis virus (JEV), and West Nile virus (WNV) have been singled out as globally important, accounting for about 100 million symptomatic cases every year [3, 5].

Although originating as a tropical and subtropical mosquito species, the invasive *Aedes aegypti* mosquitoes have been reported to appear in temperate locations due to the influence of rising global temperatures resulting from anthropogenic-induced climate change and rapid adaptation processes [6]. Found in peri-urban and urban areas, they lay eggs in natural and artificial containers, utilizing them for larvae and pupae breeding [7–9]. *Aedes albopictus,* also known as the Asian tiger mosquito, has also been found to be highly invasive, with the ability to colonize new locations with suitable environmental conditions, and ecologically flexible, and has been found in sylvatic, peri-urban, and urban habitats, making a broad spectrum of hosts available for them to feed on [10, 11]. Although *Ae. albopictus* is an opportunistic feeder with a wide spectrum of host preference compared with *Aedes aegypti*, both mosquitoes have been found to have preference for human blood, making them anthropophilic irrespective of other vertebrate hosts available in a location [12, 13]. This characteristic makes them important vectors for human-to-human and human-to-animal transmission of endemic and emerging MBDs [13]. Both mosquito species have been identified as competent vectors for several mosquito-borne pathogens and the most important vectors for DENV, CHIKV, ZIKV, YFV, and Rift Valley fever virus (RVFV) [3, 14]. *Culex pipiens* complex, regarded as one of the most important mosquito species notable in the northern hemisphere, can thrive across diverse land-use types and are competent vectors for multiple pathogens of public health interest [15]. They display environmental plasticity and can breed in several locations, including temporary or semipermanent water bodies, stagnant ponds with vegetation, water-filled tree holes, and flooded cellars [16]. Ovipositing female *Cx. pipiens* mosquitoes responsible for spread of pathogens can overwinter in caves, underground cellars, subways, and burrows and reactivate when environmental conditions are suitable [17]. *Cx. pipiens* are mainly ornithophilic in nature but can also feed on mammals, including humans when available, making them competent vectors for pathogens bridging transmission between mammals and birds by horizontal and possibly vertical transmission (VT) mechanisms [18–20]. *Cx. pipiens* are competent vectors for WNV, Usutu virus (USUV), Rift Valley fever virus (RVFV), and Sindbis virus (SINV) globally.

DENV, which consists of four major strains from the *Flavivirus* genus, is regarded as the most wide-spread arbovirus of public health importance, with about 12 million cases and over 8000 DENV-related deaths reported from 86 countries in 2024 [21–23]. All four strains of DENV are currently circulating in highly populated urban settlements between human hosts and *Aedes* mosquitoes, primarily *Ae. aegypti* species. Humans infected with any strains of DENV may have acute febrile illness, sudden skin rash, headache, and vomiting [3, 22]. Notably, several cases of DENV have been asymptomatic and not reported, which can contribute to the spread of the virus by effectively infecting mosquito vectors, and alter transmission dynamics [24]

First isolated between 1952 and 1953 in southern Tanzania, CHIKV is an alphavirus transmitted to humans by bites from infected *Aedes* mosquitoes, with autochthonous transmission of the virus already reported in 114 countries spread across subtropical and tropical parts of Africa, America, Asia, Europe, and Oceania, infecting millions of people globally to date [25]. Acute, atypical acute, severe acute, and chronic symptomatic CHIKV types have been clinically classified by the World Health Organizatio (WHO) [26]. A review by Rama et al. [27] reported that 75% of people infected with CHIKV will develop symptoms, 90% of them will have arthralgia, 88% will develop fever, and 0.3% are likely to die from the infection.

ZIKV is a single-strand RNA arbovirus from the *Flavivirus* genus and is also of interest to public health partly due to the environmental plasticity and invasive nature of the primary vector (*Ae. aegypti*), multiple transmission modes, and viral persistence in the body fluid of the infected host [22, 28]. Since its isolation in a Ugandan forest in 1947 during mosquito surveillance, the virus has been believed to become prevalent geographically in Southeast Asia by the 1960s, the Island of Yap by 2007, French Polynesia between 2013 and 2014, South America in 2015, and in 34 South and Central American countries since 2016 [29]. After 3–7 days, infected humans develop low-grade fever, rash, conjunctivitis, and muscle pains, which usually last for about 1 week [30]. Studies have also reported a strong relationship between the ZIKV-infected parent and risk of microcephaly in the first trimester [31]

YFV, a mosquito-borne arbovirus from the *Flavivirus* genus, originated from the tropical and subtropical areas of Africa and subsequently was introduced into

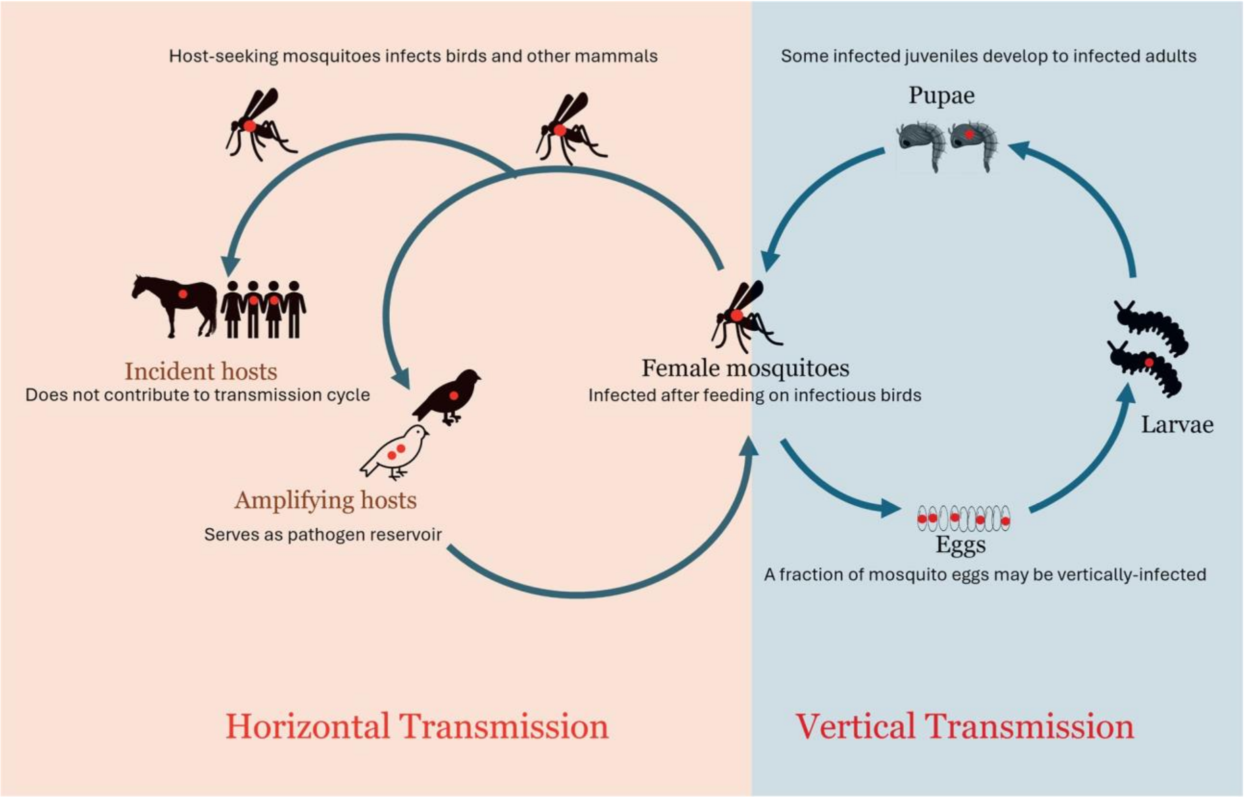

Fig. 1 Vertical transmission illustrated for mosquito-borne arboviruses (WNV illustrated). The virus sticks to the egg surface during oviposition for a trans-ovum transmission process, while the virus enters the oocyte at its developmental stage for a trans-ovarian transmission process

South America in the advent of European colonization [32]. The virus causes YFV infection, a disease with 200,000 cases and 30,000 deaths yearly, of which 90% are recorded in Africa [33]. Despite being endemic to Africa, Central America, and South America, the virus urban cycle has been successfully controlled in Brazil since 1942 [34]. However, the risk of YFV outbreak in large urban endemic areas is ever increasing, with factors such as urbanization, population structure, deforestation, and climate change playing critical roles [35]. Although most people infected with YFV do not have symptoms, some persons may have mild flu-like symptoms or high fever with jaundice and hemorrhaging from the mouth, nose, eyes, or stomach [36].

JEV is a mosquito-borne *Flavivirus* endemic in several parts of Asia and the Western Pacific, with over 3 billion people living in high-risk areas, resulting in 68,000 symptomatic cases and 13,000–20,000 deaths annually [37]. Symptoms common after being bitten by an infected *Culex* mosquito include fever, confusion, and seizures, with fatality rates as high as 30% [38].

WNV is single-strand RNA *Flavivirus* that was first isolated in the West Nile district of Uganda in 1937, with sporadic cases occurring in Africa, Eurasia, Australia, and the Middle East in the early 1900s [39]. From 1996, outbreak in humans and horses became frequent in Europe and the Middle East, afterwards spreading across North, Central, and South America after 1999 [40]. WNV can trigger WNV fever in birds, horses, humans, and other vertebrates [41]. Symptoms associated with the infection include fever, rashes, nausea, and vomiting, and in a more severe cases, neuroinvasive disease or death can occur [42]. Other arboviruses of public health interest include RVFV, St. Louis encephalitis virus (SLEV), USUV, La Crosse virus (LACV), and SINV [3].

Transmission pathways

MBDs are ideally transmitted to and from a host when a mosquito pierces through the skin of a host for a blood meal. This process of transmission is known as horizontal transmission (HT) and is assumed to be the conventional means of transmission for mosquito-borne pathogens. Another transmission process is vertical transmission (VT), which involves transmission of pathogens from adult mosquitoes to their offspring [17, 43]. VT, which is either in the form of trans-ovarian or trans-ovum

transmission (Fig. 1), has been documented for several mosquito species [44, 45].

Although there has been much emphasis on HT of arboviruses in the avian–mosquito cycle, as in WNV, and the human–mosquito cycle, as in DENV, less attention has been given to the occurrence and impact of VT. Under seasonal climatic conditions suitable for vector population and pathogen transmission, VT may supplement virus amplification during summer and provide a mechanism to infect overwintering female mosquitoes during the fall, as seen in *Cx. pipiens* mosquitoes [44].

Although arboviruses are originally maintained in a transmission cycle between the mosquito vector and vertebrate hosts naturally in an enzootic sylvatic cycle, certain factors such as climate change, deforestation, and urbanization can alter this natural cycle, creating an epizootic or rural circle, where amplification of viruses occur in domestic animals, and subsequently an urban epidemic cycle, where rapid transmission occurs between vectors and diverse kinds of hosts, including humans [46, 47].

The probability of these arboviral pathogens being successfully transmitted between vector and host is dependent on multiple drivers that are either climatic, ecological, or socioeconomic [48, 49]. These drivers determine vector availability, vector competence, vector–host interaction, and the probability of pathogen transmission, epidemic outbreak, and the endemism of MBDs.

Given the evolutionary nature of interactions and processes involved in the occurrence and transmission of arboviruses, it is expected that these drivers will alter functional traits and transmission rates, including VT, that determine outbreak dynamics. Some of these changes have been seen previously in the spatiotemporal distribution of emerging and re-emerging arborviruses of public health interest due to their intrinsic ability to thrive in multiple hosts and vectors, triggering sporadic changes in their transmission cycle [23]. In an attempt to control and eliminate MBDs, mosquito population control programs are initiated to limit and subsequently eliminate outbreaks of MBDs. However, certain factors such as VT, which have a latent but potentially strong effects, are neglected when analyzing failed control efforts.

Lequime et al. [50], who previously analyzed the historical trend of scientific investigations on experimental and vertical transmission in mosquitoes, revealed that, although the extent and significance of VT are still debated, arboviral emergence stimulated an increase in VT research, while recent laboratory essays enhanced VT detection. Ferreira-de-Lima et al. [51] reported a correlation between vertical transmission and endemism of DENV, especially in South American countries, and cited a lack of studies as a possible reason for the gap in reporting VT from other Dengue-endemic areas, such as Africa. Janjoter et al. [52] reviewed transovarial transmission of mosquito-borne arboviruses and identified factors affecting transovarian transmission, the potential implications, mosquito antiviral defense mechanisms, and strategies to control mosquito-borne arboviruses.

Here, we designed a systematic review to evaluate studies that were conducted to identify the presence or impact of VT of arboviruses in mosquito populations with an emphasis on the study type, mosquito species, and arbovirus.

In this systematic review, we evaluated studies aimed at identifying the presence or impact of VT of arboviruses in the mosquito population, with a focus on study type, mosquito species, and arbovirus genus. The objective of the review is to build on previous reviews, identify similarities and research gaps, present the current state of the art in investigating VT of arboviruses in mosquito populations, and explore their incorporation into mathematical models for various mosquito-transmitted arboviruses.

Methods

Literature search and data collection

We adopted the Preferred Reporting Items for Systematic Reviews and Meta-analyses (PRISMA) approach for our systematic review article search to aid article selection process (Fig. 2). The Web of Science database was used to conduct an extensive search for relevant documents. Terms considered relevant were used to construct a string that was used for the search and had 794 hits: ("vertical transmi*" OR "transovaria*" OR "transovu*" OR "transegg") AND ("mosquito-borne disease*" OR "mosquito borne disease*" OR " vector borne disease*" OR " vector-borne disease*" OR "Dengue*" OR "DENV*" OR "Chikungunya*" OR "CHIKV*" OR "Zika*" OR "ZIKV*" OR "West Nile virus*" OR "WNV*" OR "Sindbis virus*" OR "SINV*" OR "yellow fever virus*" OR "YFV*"). Studies from 1950 to 2024 identified from the search were screened for inclusion and exclusion. An additional 43 studies identified from screening references were also added. Proceeding papers, editorial materials, letters, descriptive studies, and review articles were excluded. Full articles that were not related to mosquito-borne diseases, not focused on arbovirus, not related to transoval or trans-ovarian transmission, and duplicated studies were also removed. In all, 175 documents were finally selected and used for the systematic review.

Articles were classified into categories using a template designed to extract the relevant information. A study summary table was built to assess and present information extracted from each article. Relevant information

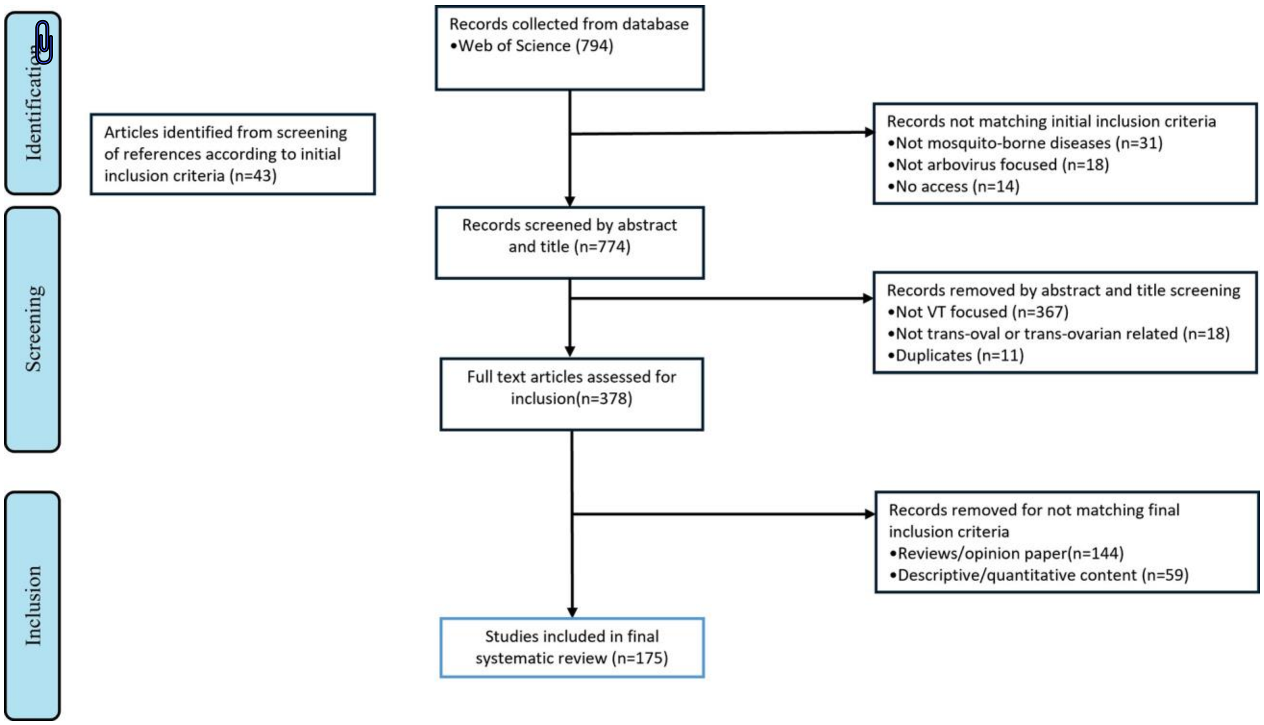

Fig. 2 Preferred Reporting Item for Systematic Reviews and Meta-analysis (PRISMA) styled flow diagram of literature search process. Articles identified from the Web of Science database and references of relevant journal articles were screened

related to study type, mosquito species, arbovirus genus, location, and reported VT efficiency were presented in a table (*Summary Table* in Supplementary Material). Articles included publications focused on MBDs where either modeling or field or laboratory analysis was conducted. Spatial distribution of the locations where studies on vertical transmission have been conducted are shown in Fig. S1 in the Supplementary Material.

Findings from literature search

Diversity of mosquito species and pathogen species

A summary table was generated from the 175 full articles used containing details extracted from each study (*Summary Table* in Supplementary Material). In regard to the study approach, 24 studies applied modeling approaches, 62 conducted only laboratory analyses, 86 carried out only field investigations, and 3 studies conducted both laboratory analyses and field investigation. Details on mosquito species, arbovirus genera, and study types have been summarized (Fig. 3).

VT has been widely studied and identified in several mosquito species. *Ae. aegypti* and *Ae. albopictus* mosquitoes, which are regarded as invasive and urban mosquitoes, were the most widely studied, with 146 studies in total (*Summary Table* in Supplementary Material; Fig. 3). This was expected, as *Aedes* mosquitoes have been reported on every continent and are vectors of numerous pathogens of public health interest. Other *Aedes* mosquitoes that were found in our study are *Ae. vexans*, *Ae. vigilaz*, *Ae. camptorhynchus*, *Ae. japonicus*, *Ae. triseriatus*, and *Ae. ochraceous*. To highlight the importance of invasive *Aedes* species in mosquito-borne disease epidemiology and VT, 81 studies were either related to the identification or implication of VT of DENV in *Aedes* mosquitoes, 32 studies were related to ZIKV, and 20 of them investigated the potentials of VT of CHIKV in *Aedes* mosquito population. Also, six studies investigated the potential VT of YFV in *Aedes* mosquitoes. Others appeared in modeling studies.

Although *Culex* mosquitoes were widely studied according to the review, *Cx. pipiens* complex was the most prominent, appearing in 13 studies; 12 studies were all related to identifying VT of WNV, while 1 was on SINV (*Summary Table* in Supplementary Material). Other *Culex* mosquitoes that were identified were *Cx. poicilpes* in a modeling study by Favier et al. [53] related to RVFV. *Cx. annulirostris,* and *Cx. globocoxitus* were also in another modeling study by Koolhf et al. [54] for RRV. *Cx. quinquefasciatus* appeared in five studies for WNV, one study for SLEV, and one study for ZIKV. *Cx. tarsalis* was investigated for VT of WNV in three

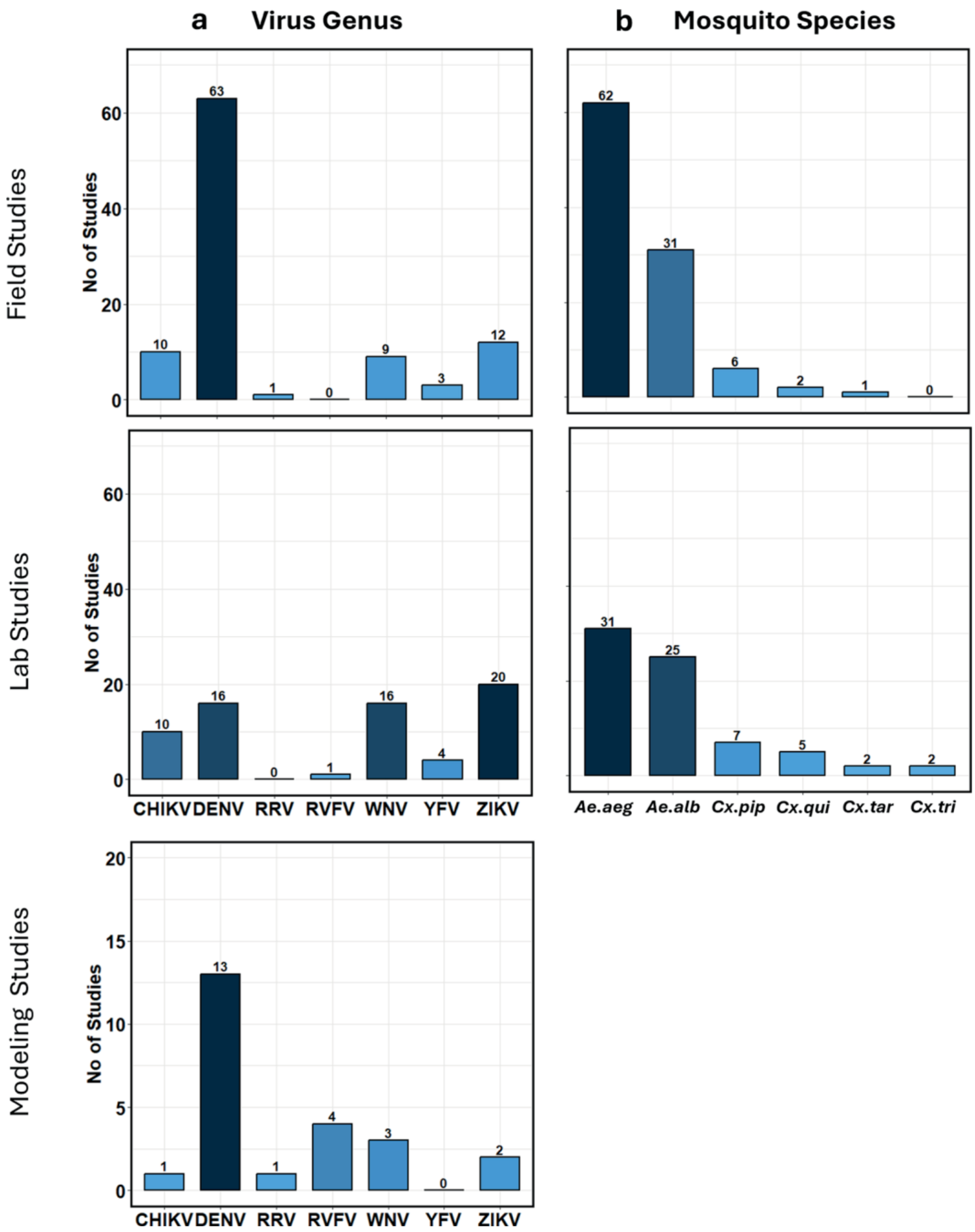

Fig. 3 Diversity of viruses and mosquito species found in the study. **a** Arbovirus genus and the number of studies reported for field, lab, and modeling studies. DENV, CHIKV, ZIKV, WNV, RVFV, and YFV had the most records. **b** Number of studies and the diversity of mosquito species reported in field and laboratory studies. *Ae. aegypti*, *Ae. albopictus*, *Cx. pipiens*, *Cx. quinquefasciatus*, *Cx. tarsalis*, and *Culex tritaeniorhynchus* were the most reported

studies, while *Culex stigmatosoma* and *Culiseta annulata* both appeared in studies related to VT of WNV. *Cx. tritaeniorhynchus* also appeared in two studies for VT of WNV [55, 56]. Other *Culex* mosquitoes appeared a few times in the studies (*Summary Table* in Supplementary Material).

Occurrence, rate, and impact of vertical transmission

Investigating the occurrence of VT of arboviruses within different mosquito species and the impact of VT on the dynamics of mosquito-borne arboviruses were the two foci of our review. Important mosquito species most identified to support VT were *Ae. aegypti*, *Ae. albopictus*, *Ae. vexans*, *Cx. pipiens*, *Cx. tarsalis*, *Cx. quinquefasciatus*,

and *Cx. tritaeniorhynchus*, while pathogens identified to be transmitted vertically were DENV, ZIKV, WNV, CHIKV, YFV, SINV, RRV, and MAYV. VT was found to occur in both natural and laboratory settings (*Summary Table* in Supplementary Material). In studies where reports of VT rates were available, VT was found to occur naturally in 47 field studies, while 33 laboratory studies reported VT experimentally (*Summary Table* in Supplementary Material). VT was also found to occur sparingly naturally and in laboratory conditions in other mosquito species and for several arboviruses (*Summary Table* in Supplementary Material). For VT detection, the reverse transcription polymerase chain reaction (RT-PCR) method was commonly used across studies evaluated.

While it has been argued that the effect of VT on infection persistence is negligible due to its low infection rate ranging from 1–4%, VT could become a significant driving factor when regular pathogen amplification occurs in suitable reservoir host populations, which has been reported in DENV cases and could potentially apply to other arboviruses such as WNV [57]. Vertical transmission rates reported have been extracted and documented (*Summary Table* in Supplementary Material). Statistically, we presented only the minimum infection rate (MIR) (Figs. 4, 5), which is widely used to report virus infection rate and represents the ratio of number of positive pools to the total number of specimens tested [58].

Certain environmental factors affect the rate of VT. Studies by Taghikhani et al. [59] and Chitnis et al. [60] identified the effect of seasonal fluctuations in temperature and rainfall on VT and disease outbreak rates. Infection transmission from onset and pathogen spread, establishment of endemism, and disease prevalence were all linked to VT [51, 59–61].

Most of the studies that applied mathematical models investigated the impact of VT on pathogen transmission dynamics, which differs across studies and location. Yuan et al. [61], Abidemi et al. [62], Abdullah et al. [63], Alves

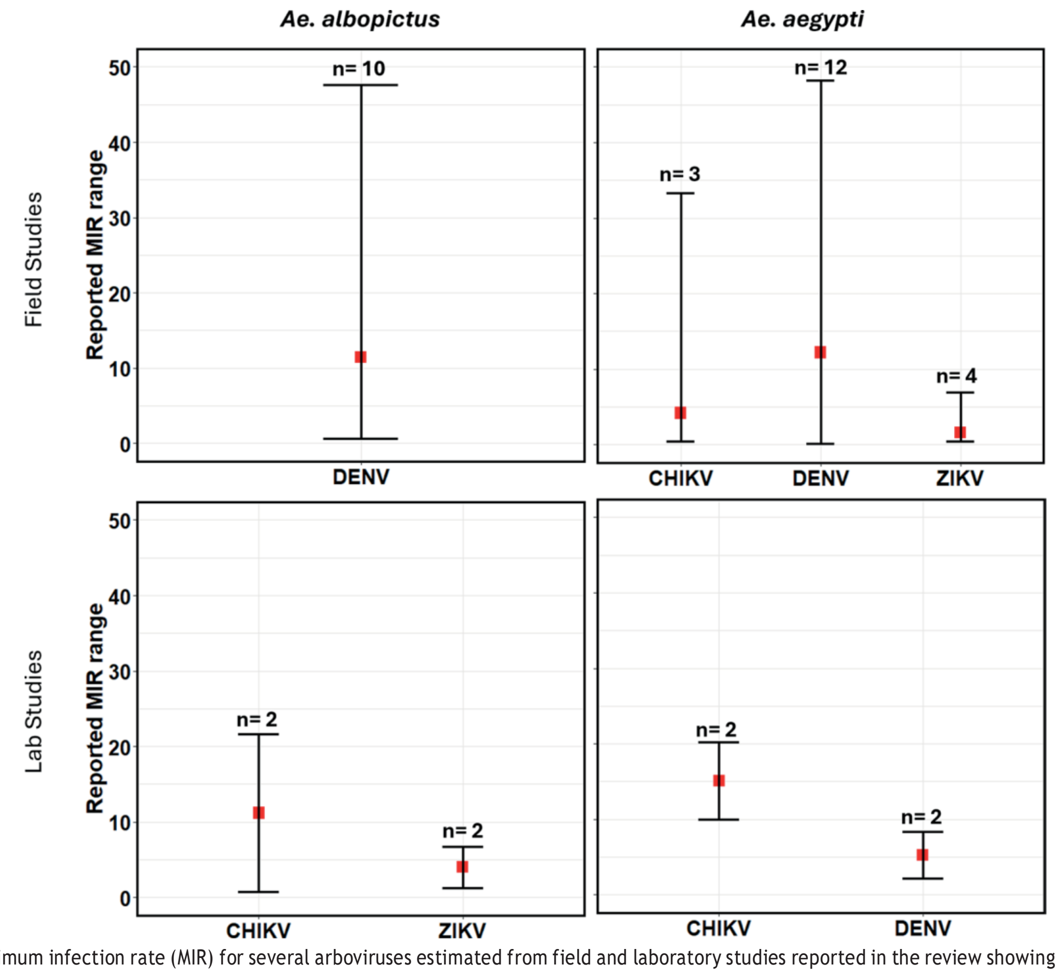

Fig. 4 Minimum infection rate (MIR) for several arboviruses estimated from field and laboratory studies reported in the review showing the range and median for *Ae. albopictus and Ae. aegypti* mosquitoes

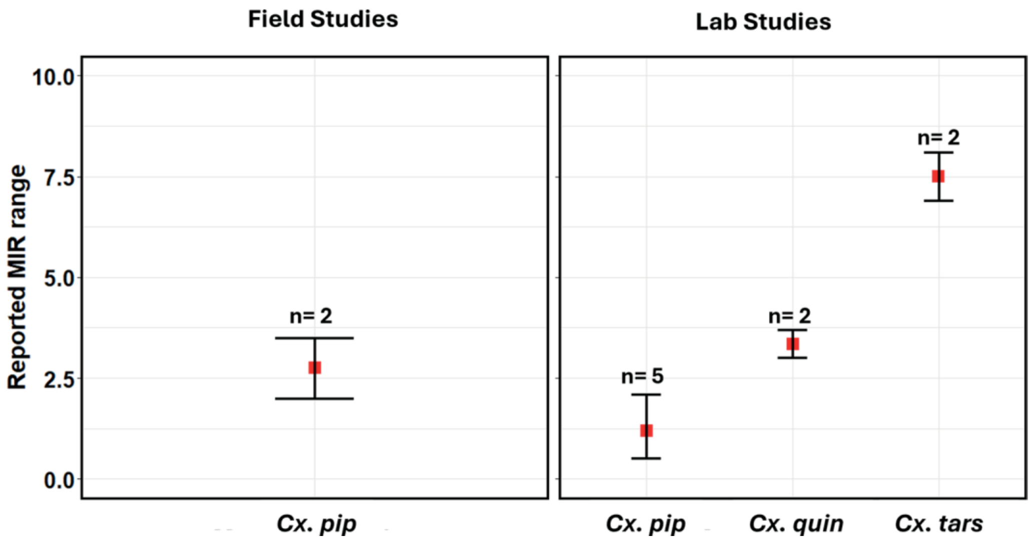

Fig. 5 Minimum infection rate (MIR) for West Nile virus estimated from field and laboratory studies reported in the review showing the range and median for *Cx. pipiens*, *Cx. quinquefasciatus*, and *Cx. tarsalis* mosquitoes

et al. [64], Murillo et al. [65], Blayneh et al. [66], Aliyu et al. [67], and Fan et al. [68] all investigated and established relationships between VT and infection transmission rate, disease prevalence, and control measures using mathematical modeling approaches. Similarly, the impact of VT on the rate of pathogen spread was demonstrated in a mathematical model by Wang et al. [69]. Also, Wang et al. [70] reported that, even when the basic reproductive number is less than or close to 1, an increase in VT will lead to a disease outbreak for CHIKV. In contrast, Cheng et al. [71] investigated probable causes of DENV outbreak in Guangzhou city, China, and reported that the time of imported cases, precipitation, and temperature changes were more important factors than VT. A study by Cavalerie et al. [72] also could not establish a relationship between VT and RVFV persistence over several years in Mayotte Island.

Drivers of vertical transmission

MBDs have continued to spread to new areas where they were previously absent or had been eliminated. Some of these diseases break out sporadically and slow down, while some have become endemic, causing substantial economic loss and have become a burden to public health locally, regionally, and globally. The rate of emergence and spread of MBDs have been linked to several factors, most of which have been broadly studied, spanning across several mosquito species, pathogen genera, and geolocation. However, limited studies have been conducted to understand the driver MBDs in the context of VT, as most of these studies have been focused on HT. Generally, factors that affect HT and VT are either intrinsic or extrinsic. Janjoter et al. [52] listed virus strain, mosquito species, gonotrophic cycle, blood meal, and climate, notably temperature, as determinants of the number of female mosquitoes that transmit viruses vertically. We explored these drivers and their attributes to understand their status regarding the state of the art and how they are related to studies in our review. Viral trait is a key determinant of VT. Attributes that relate to this factor include the types of virus strain, infection rate, virulence, survival and replication of viruses in mosquitoes, and persistence of viruses in an egg after infection [52, 73, 74]. Vectoral trait, which relates to the nature of vector, is another important factor. This includes the nature of vector species and strain, sensitivity of functional trait to the environment, progeny fitness and its effect on development rate, vector behaviors such as hibernation and overwintering activities that tend to support virus survival, and persistence through VT in the absence of HT [75–81]. The impact of climatic factors such as temperature, humidity, and precipitation on habitat availability, breeding success, and subsequent population increase directly would possibly impact the rate of vertical transmission [60, 82]. Ecological factors, which include ecosystem modification anthropogenically and naturally through disturbances, can introduce fragmented habitats, creating an enabling environment for vector population to thrive, increasing vector–host interaction and increasing HT [83] and possibly VT. Other anthropogenic activities such as vector population control, infection control, and vaccination can alter the rate of pathogen outbreak and either decrease or increase the rate of transmission in a location. The impact of

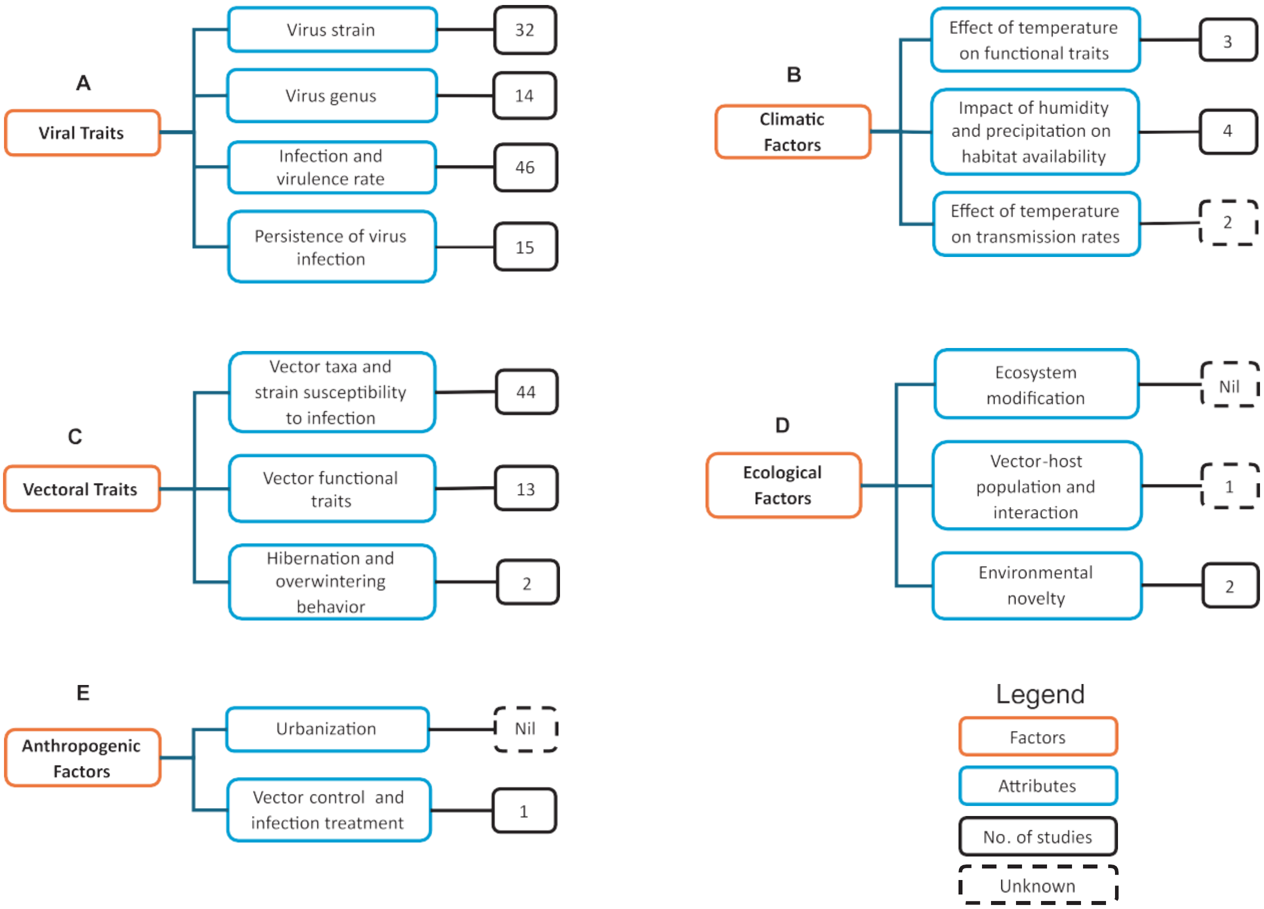

Fig. 6 Drivers identified to support vertical transmission in mosquito-borne disease epidemiology. **A** Viral traits consist of virus strain, virus genus, rate of infection, and virulence and persistence of viruses after infection. **B** Vectoral traits consist of vectoral species and a strain's susceptibility to infection, vector functional traits, and vectoral behaviors that relate to hibernation and overwintering. **C** Climatic factors, which include the sensitivity of functional traits to temperature, impact of humidity and precipitation on habitat availability, and effect of temperature on transmission rates. **D** Ecological factors, which consist of attributes related to ecosystem modification, vector-host interaction, and habitat heterogeneity. **E** Anthropogenic factors, which include urbanization and activities related to vector control and treatment of infected humans and animals

viral traits on VT was the most studied, with 107 studies related to them in total. This was closely followed by vectoral traits, with 59 studies. Interestingly, factors relating to climate have been neglected, with only nine studies. The impact of ecological and anthropogenic factors are the least studied factors, with three studies and one study, respectively. This represents a clear research gap in the state of the art with regards to the drivers of VT (Fig. 6).

Vertical transmission rate variation

Vertical transmission rate or progeny infection rate were reported either as minimum infection rate (MIR), filial infection rate (FIR) or percentage of infected progenies. These have been summarized and reported statistically showing the range and median for each mosquito species and arbovirus genus (Figs. 4, 5). For *Ae. albopictus* mosquitoes, vertical transmission rate estimated from MIR for DENV were between 2.2 and 47.6 in the field. For CHIKV, MIR values ranged between 0.76 for field studies. For ZIKV, MIR values reported were between 1.3 and 6.7 in the lab. For *Ae. aegypti* mosquitoes, MIR values reported for DENV ranged between 0.18 and 48.2 for field studies, while values between 2.13 and 8.33 were reported for lab studies. For ZIKV, the range of values reported were between 0.45 and 6.9 in the field. For CHIKV, values reported were between 0.45 and 33.3 for field studies while values reported for lab studies were between 10 and 20.2. For *Cx. pipiens* mosquitoes, field values for WNV ranged between 2.0 and 3.5 while laboratory experiments reported values between 0.52 and 2.1. For *Cx. tarsalis*, values reported for laboratory studies were between 6.9 and 8.1. For *Cx. quinquefasciatus* mosquitoes, laboratory experiments reported values between

3.0 and 3.7. Clearly, DENV had more MIR reported in field studies than any other arbovirus, with relatively high values for *Aedes* mosquitoes. This was expected as *Aedes* mosquitoes have long been designated as the competent vectors of DENV. Relatively high MIR were also reported for CHIKV in the field and laboratory studies in *Aedes* mosquitoes. MIR reported in *Culex* mosquitoes were not as high as those reported for *Aedes* mosquitoes. Regardless, the occurrence of VT within their population is concerning. *Cx. tarsalis* had the highest range in the lab closely followed by *Cx. pipiens* in the field which is interesting because *Cx. pipiens* has been long established as the common house mosquito in the northern hemisphere.

Although MIR reported seem to vary within studies, spatially between locations and across vector and virus strain, we could not ignore this result due to the sensitivity of VT to the seasonality of temperature which could support pathogen replication in mosquitoes [59]. Edillo et al. [84] reported variation in VT rate across different seasons with MIR rates ranging between 0 for wet season and as high as 48.8 in mid-dry season in Cebu City, Philippines, signaling the impact of climatic conditions. Changes in mosquito behavior like diapause and overwintering in response to climate dynamics are inevitable. Zhang et al. [85] reported that infected *Ae. albopictus* eggs were still able to hatch and transmit WNV to their progenies after termination of diapause. Interestingly, in a study by Guo et al. [86] survival and replication of DENV in *Ae. albopictus* eggs were more active in non-diapausing eggs, reducing VT success rate. The effect of virus strain is also an important responsible for VT rate variation. In a study by Freier et al. [87] to test the ability of 17 strains of *Aedes* mosquitoes to vertically transmit DENV using all 4 DENV strains, DENV-1 was transmitted vertically by 11 of the mosquito strains representing 8 different species. Similarly, Rosen et al. [88] recorded high transovarial transmission of DENV-1 strain and low transmission rates for DENV-3 in a study where all 4 DENV strains were used. Velandia-Romero et al.[89] evaluated transovarial transmission of DENV in larvae and pupae of *Ae. aegypti* and found more juvenile infected with DENV-1 than other DENV strains. Additionally, Mitchell [90] investigated the ability of three strains of *Ae. albopictus* to vertically transmit DENV-1 and DENV-4 strains. It was observed that 7 pools out of 60 were positive for DENV-1 while only 1 pool out among 121 were positive for DENV-4. Findings from the review supported the view that important functional traits of mosquitoes such as extrinsic incubation period and gonotrophic cycle can determine VT rates. Manuel et al. [91] observed that longer extrinsic incubation time and fewer gonotrophic cycle supports VT success of ZIKV in *Ae. aegypti* mosquitoes, indicating the influence of longer egg development time in gestating female mosquitoes. Additionally, relatively high VT rates were observed in the second gonotrophic cycle. This was similar to results from a study by Zhu et al. [92] where VT rates were significantly higher from the second gonotrophic cycle. Progeny fitness was also reported to affect VT success rate. In a study by Joshi et al. [81] to evaluate the persistence of DENV-3 virus through transovarial transmission in *Ae. aegypti* mosquitoes, it was observed that larval duration of vertically infected juvenile mosquitoes increased significantly compared with uninfected control juveniles, signaling an effect of vertically transmitted infection on progeny development rate. Similarly, Turell et al.[79] reported that pupae infected with RVFV failed to emerge as adult, a phenomenon which may be responsible absence of VT from the study. Regardless of the variations, these findings are important given that the probable minimum rate of VT that could alter transmission dynamics of mosquito-borne arbovirus like DENV ranges between 4–20 [93]. The reported VT rates are very useful to quantify mosquito species and strain susceptibility to certain arboviruses, which can also be incorporated into design and parametrization of mathematical models that account for VT in mosquito population.

Global change, vertical transmission and MBDs epidemiology

The emergence and spread of MBDs have been consistently linked to global change driven by a combination of environmental and socioeconomic processes. Although recent research has often focused on the impact of climate change on vector population dynamics, vector competence, vectoral capacity, pathogenesis and pathogen transmission, other processes linked to global change have been identified to have an interactive effect on MBDs [94]. Environmental and socioeconomic processes have been factored into risk determination models that have been successfully used to explain the distribution of zoonotic diseases [95]. These processes eventually drive HT and VT of MBDs. Some of the drivers identified to support VT and subsequently impact mosquito-borne disease epidemiology were linked to processes and activities attributed to global change.

Changes in local or global climate patterns, which are one of the most important indicators of global change, have direct and indirect impacts on VT [59]. Generally, temperature has been identified as a determinant of incidence and severity of MBDs outbreak by altering the processes of pathogen evolution, selection and transmission [96]. Functional traits of mosquitoes are sensitive to temperature [97]. Favorable temperature can alter mosquito development rate, shortening breeding

time and increase population density within a short time [98]. Also, an increase in temperature could lead to an increased biting rate, shortening the gonotrophic cycle and enhancing contact rate which increases possibilities of infection and cross infection between vector and host [96]. Changes in climate pattern may give rise to extended breeding season allowing mosquito population to thrive and pathogen transmission persist beyond favorable which increases the likelihood of VT to support pathogen survival in the absence of competent amplifying hosts [99]. Changes in precipitation pattern could support availability of mosquito breeding sites, enhance population density and support VT. Result of interaction between vectors and pathogens are influenced by convergent evolution and ecological factors which can induce pressure and trigger arboviruses to develop mechanisms of VT [75]. Genetic signatures of pathogens that trigger MBDs may exhibit distinct responses to variability of climate variables like temperature, triggering geographic variation in terms of outbreak [100] and will have an overall in affect rate of HT and VT. Ecosystem modification by humans triggered by deforestation, urbanization and intensive agriculture can shift geographical distribution of vectors, reservoir host of pathogens, create suitable environmental conditions for better vector-host interaction and increase availability of vector breeding sites The combined result from these would increase vector-host interaction. Vector- host interaction has been reported to affect VT success in a study by Edillo et al. [84] where juvenile mosquitoes collected from households had higher VT rates than those collected in the field, possibly due to increased interaction between mosquitoes and infected humans. Diouf et al. [101] reported variation in VT rate across breeding sites and habitat landcover type. Additionally, Rohani et al. [102] identified the impact of breeding site on VT rate where *Ae.albopictus* mosquitoes which preferred shaded areas had more infected larvae than *Ae. aegypti* larvae which preferred clear water.

The effect of global change has triggered the introduction of invasive mosquito vector species and vector strains in certain regions. Invasive mosquito species such as *Ae. aegypti*, *Ae. albopictus* and the common house mosquito in Europe *Cx. pipiens* which have been identified as competent vectors of several arboviruses of public interest possess high susceptibility of pathogen infection and replication. They have also been found to possess the ability to naturally transmit these viruses horizontally to competent hosts and vertically to their progenies [17, 103]. The co-existence of vectors and competent amplifying host increases the likelihood of cross-infection and subsequently VT from vector to their progeny [84]. Migration of competent hosts of several arboviruses driven by global change is a determinant of host population distribution. As a result of these migrations or prior population availability, settlements with high concentration of human population density have recorded sustained outbreak of arboviral infections such as DENV, CHIKV and ZIKV, some of which have been attributed to the presence of VT [84, 103]. Avian-mosquito circulated arboviruses such as WNV have also been documented to be introduced to new locations during seasonal migration of birds over long distances, which are subsequently maintained and amplified by competent residential birds [104]. Outbreaks have also persisted in locations where competent amplifying host birds and vector mosquito persist in favorable environmental conditions, with the presence of VT not ruled out.

Although often neglected, our study has been able to reveal the potential importance of VT in emergence, persistence and spread of MBDs. VT has been presumed to be an important factor in the long-term persistence of several mosquito-borne arboviruses in a vector-host cycle without the necessity of virus re-introduction, a process which sabotages control efforts and causing outbreaks which may be expensive and impossible to control and could increase infection prevalence [65, 77]. It has also been reported that although VT alone may not establish endemism of a disease, it can increase prevalence and endemic level of disease on vector and host population which can result to a pathogen transmission regime [66, 69]. Studies have also reported that an increase in VT rate could lead to a disease outbreak even when basic reproduction number is below or close to 1 [70]. Also, VT could support endemism where certain conditions like the availability of a permanent pathogen reservoir, sufficient inter-site rainfall variability and host movement to locations with favorable environmental conditions [53]. In a study by Thongrungkiat et al. [105], the beginning of a Dengue season was preceded by a high VT isolation in field collected *Ae. aegypti* larvae, denoting the impact of VT on rates of DENV outbreak and the significance of VT as an epidemiological tool for potential application of mosquito control intervention.

Cases of co-infection by VT were reported in several studies, signaling the possibilities for the VT of multiple arbovirus genera and strains. Teixeira et al. [106] reported co-infection of DENV and CHIKV in mosquito larvae reared from *Ae. aegypti* eggs collected from a city of Vitória da Conquista, Bahia, Brazil. Similarly, Granger Neto et al. [107] detected co-infection of vertically transmitted DENV and CHIKV of *Ae. aegypti* larvae in Brumado, Bahia, Brazil. Interestingly, both cases of co-infection were reported in the state of Bahia, one of the most affected with the highest number of reported

DENV in Northeastern Brazil [108]. Cecílio et al. [109] detected co-infection of vertically transmitted DENV-1 and DENV-2 in larvae reared from eggs collected from Pampulha, Belo Horizonte, Brazil, which is a city with the second highest number of DENV cases in Belo Horizonte. Pessanha et al. [110] isolated DENV-2 and DENV-3 in *Ae. aegypti* larvae also collected in Belo Horizonte, Minas Gerais, Brazil. DENV-1 was also reported to co-infect juvenile mosquitoes collected from households in a study by Edillo et al. [84] in Philippines. Similarly, Velandia-Romero et al. [89] reported that DENV-1 was found to co-infect juvenile with DENV-2, DENV-3 and DENV-4 more than any other strain in Colombia. All cases of co-infection reported were from South America where DENV, CHIKV and ZIKV are endemic. Co-infection of DENV-1 with other DENV strains was also reported in 3 studies above signaling the epidemic significance of DENV-1 strain.

From the graphical representation for global distribution of studies of VT in arboviruses, South America where several arboviruses such as DENV, CHIKV and ZIKV are endemic have the highest number of studies (Fig. S1 in Supplementary Material). This finding aligned with the submission of Ferreira-de-Lima et al. [51] about a relationship between VT and endemism of several arboviruses of public health interest in South American countries. Interestingly, WNV which has been endemic in Europe and North America had a total of 5 studies conducted in the United States but only 1 study each in Austria and Italy which is interesting because WNV VT was reported in *Cx. pipiens* mosquito population in field studies by Rudolf et al. [77] and Kolodziejek et al. [20] in Austria. This clearly shows a research gap existing in Europe.

Conclusions

The effect of global change has been evident in the emergence, prevalence and spread of MBDs. About 73% of emerging and remerging MBDs are caused by arboviruses including DENV, CHIKV, ZIKV, YFV, JEV, and WNV. These arboviruses can be transmitted horizontally between mosquitoes and competent host during blood-feeding or vertically between female mosquitoes and their progenies. Although studies have focused on the horizontal transmission pathway, vertical transmission of arboviruses of interest have been reported in several studies. We conducted a systematic review to evaluate the state-of-the-art and relevance of vertical transmission of arboviruses in mosquito-borne disease epidemiology.

In our study, VT was confirmed for DENV, CHIKV, ZIKV, WNV, SINV, YFV and MAYV more often. We also identified studies that established relationships between VT and infection rates, disease prevalence and efficacy of outbreak control efficacy. Viral traits, vectoral traits, climatic factors, ecological factors and anthropogenic factors were identified as potential drivers of vertical transmission in a mosquito population, highlighting the status and relationship of these factors in terms of number of studies relating to each of them. The impact of ecological and anthropogenic factors on VT of MBDs are still poorly explored. Also, we were able to establish the state-of the art on studies related to VT in mosquito population and the relevance of VT of arboviruses in mosquito-borne disease epidemiology, highlighting arboviruses of public health interest transmitted vertically and their occurrence naturally and experimentally. We also highlighted mosquito species that support VT of arboviruses in their population, potential drivers of VT and the impact of global change in driving VT in mosquito population.

With the presence of VT established in multiple mosquito species population for several arboviruses of public health interest, it is recommended that similar consideration and attention be accorded to vertical and horizontal transmission mechanisms in studies relating to mosquito-borne diseases dynamics. This would enable researchers and policy makers to develop robust tools and policies that can efficiently help in the control and elimination of MBDs.

Supplementary Information

The online version contains supplementary material available at https://doi.org/10.1186/s13071-025-06761-8.

Additional file 1.

Acknowledgements

Not applicable.

Author contributions

OCM and CB conceptualized the study. OCM and SMT designed the systematic review process. OCM processed the data, prepared all results and figures, wrote the first draft and prepared the supplementry files. All authors were involved in reviewing and editing the manuscript.

Funding

Open Access funding enabled and organized by Projekt DEAL. OCM and SMT were funded by the Bavarian State Ministry of the Environment and Consumer Protection and the Bavarian State Ministry of Health and Care through the BayByeMos project (AP-2411-PN 21-14-V3-D22827/2022) within the Joint Project "Climate Change and Health II" (VKG II). This publication was funded by the Open Access Publishing Fund of the University of Bayreuth and Chair of Biogeography.

Availability of data and materials

No datasets were generated or analysed during the current study.

Declarations

Ethics approval and consent to participate

Not applicable.

Consent for publication
Not applicable.

Competing interests
The authors declare no competing interests.

Received: 7 November 2024 Accepted: 11 March 2025
Published online: 09 April 2025

References
1. Paz S. Climate change: a driver of increasing vector-borne disease transmission in non-endemic areas. PLoS Med. 2024;21:e1004382. https://doi.org/10.1371/journal.pmed.1004382.
2. Ferguson NM. Challenges and opportunities in controlling mosquito-borne infections. Nature. 2018;559:490–7. https://doi.org/10.1038/s41586-018-0318-5.
3. Madewell ZJ. Arboviruses and their vectors. South Med J. 2020;113:520. https://doi.org/10.14423/SMJ.0000000000001152.
4. Manouana GP, Sarah-Matio EM, Hellhammer F, Zahouli J, Tapé ASB, Biré YN, et al. Ecology of arboviruses and their potential mosquito vectors in Benin, Côte d'Ivoire and Gabon: a mini review. Front Trop Dis. 2024;5:1355778. https://doi.org/10.3389/fitd.2024.1355778.
5. Carvalho VL, Long MT. Perspectives on new vaccines against arboviruses using insect-specific viruses as platforms. Vaccines. 2021;9:263. https://doi.org/10.3390/vaccines9030263.
6. Facchinelli L, Badolo A, McCall PJ. Biology and behaviour of *Aedes aegypti* in the human environment: opportunities for vector control of arbovirus transmission. Viruses. 2023;15:636. https://doi.org/10.3390/v15030636.
7. David MR, Maciel-de-Freitas R, Petersen MT, Bray D, Hawkes FM, Fernández-Grandon GM, et al. Aedes aegypti oviposition-sites choice under semi-field conditions. Med Vet Entomol. 2023;37:683–92. https://doi.org/10.1111/mve.12670.
8. Ferede G, Tiruneh M, Abate E, Kassa WJ, Wondimeneh Y, Damtie D, et al. Distribution and larval breeding habitats of *Aedes mosquito* species in residential areas of northwest Ethiopia. Epidemiology health. 2018;40:e2018015 https://doi.org/10.4178/epih.e2018015.
9. Dom NC, Ahmad AH, Ismail R. Habitat characterization of *Aedes sp*. breeding in urban hotspot area. Procedia Soc Behav Sci. 2013;85:100–9. https://doi.org/10.1016/j.sbspro.2013.08.342.
10 Westby KM, Adalsteinsson SA, Biro EG, Beckermann AJ, Medley KA. *Aedes albopictus* populations and larval habitat characteristics across the landscape: significant differences exist between urban and rural land use types. Insects. 2021;12:196. https://doi.org/10.3390/insects12030196.
11. Schaffner F, Medlock JM, Van Bortel AW. Public health significance of invasive mosquitoes in Europe. Clin Microbiol Infect. 2013;19:685–92. https://doi.org/10.1111/1469-0691.12189.
12. Cebrián-Camisón S, Martínez-de la Puente J, Figureuerola J. A literature review of host feeding patterns of invasive *Aedes* mosquitoes in Europe. Insects. 2020;11:848. https://doi.org/10.3390/insects1112084.
13 Pereira-dos-Santos T, Roiz D, Lourenço-de-Oliveira R, Paupy C. A systematic review: is *Aedes albopictus* an efficient bridge vector for zoonotic arboviruses? Pathogens. 2020;9:266. https://doi.org/10.3390/pathogens9040266.
14. Leta S, Beyene TJ, De Clercq EM, Amenu K, Kraemer MU, Revie CW. Global risk mapping for major diseases transmitted by *Aedes aegypti* and *Aedes albopictus*. Int J Infect Dis. 2018;67:25–35. https://doi.org/10.1016/j.ijid.2017.11.026.
15. Haba Y, McBride L. Origin and status of *Culex pipiens* mosquito ecotypes. Curr Biol. 2022;32:R237–46. https://doi.org/10.1016/j.cub.2022.01.062.
16. Liu X, Baimaciwang Y, Wu H, Pengcuociren G, Guo Y, Liu Q, et al. Breeding site characteristics and associated factors of *Culex pipiens* complex in Lhasa, Tibet, PR China. Int J Environ Res Public Health. 2019;16:1407. https://doi.org/10.3390/ijerph16081407.
17 Nelms BM, Macedo PA, Kothera L, Savage HM, Reisen WK. Overwintering biology of *Culex* (Diptera: Culicidae) mosquitoes in the Sacramento valley of California. J Med Entomol. 2013;50:773–90. https://doi.org/10.1603/me12280.
18. Hamer GL, Kitron UD, Goldberg TL, Brawn JD, Loss SR, Ruiz MO, et al. Host selection by *Culex pipiens* mosquitoes and West Nile virus amplification. Am J Trop Med Hyg. 2009;80:268.
19. Blom R, Krol L, Langezaal M, et al. Blood-feeding patterns of *Culex pipiens* biotype pipiens and pipiens/molestus hybrids in relation to avian community composition in urban habitats. Parasit Vectors. 2024;17:95. https://doi.org/10.1186/s13071-024-06186-9.
20. Kolodziejek J, Seidel B, Jungbauer C, Dimmel K, Kolodziejek M, Rudolf I, et al. West Nile virus positive blood donation and subsequent entomological investigation, Austria, 2014. PLoS One. 2015;10(5):e0126381 https://doi.org/10.1371/journal.pone.0126381.
21. European Centre for Disease Prevention and Control. Dengue worldwide overview. European Centre for disease prevention and control. https://www.ecdc.europa.eu/en/dengue-monthly. Accessed 2 Sep 2024.
22 Chakhtoura N, Hazra R, Spong CY. ZIKV virus: a public health perspective. Curr Opin Obstet Gynecol. 2018;30:116–22. https://doi.org/10.1097/GCO.0000000000000440.
23. Laiton-Donato K, Guzmán-Cardozo C, Peláez-Carvajal D, Ajami NJ, Navas MC, Parra-Henao G, et al. Evolution and emergence of mosquito-borne viruses of medical importance: towards a routine metagenomic surveillance approach. J Trop Ecol. 2023;39:e13. https://doi.org/10.1017/S0266467423000019.
24. Duong V, Lambrechts L, Paul RE, Ly S, Lay RS, Long KC, et al. Asymptomatic humans transmit dengue virus to mosquitoes. Proc Natl Acad Sci. 2015;112:14688–93.
25. Bettis AA, L'Azou Jackson M, Yoon IK, Breugelmans JG, Goios A, Gubler DJ, et al. The global epidemiology of Chikungunya from 1999 to 2020: a systematic literature review to inform the development and introduction of vaccines. PLoS Negl Trop Dis. 2022;16:e0010069. https://doi.org/10.1371/journal.pntd.0010069.
26. Suhrbier A. Rheumatic manifestations of Chikungunya: emerging concepts and interventions. Nat Rev Rheumatol. 2019;15:597–611. https://doi.org/10.1038/s41584-019-0276-9.
27. Rama K, de Roo AM, Louwsma T, Hofstra HS, do Amaral SG, Vondeling GT, et al. Clinical outcomes of chikungunya: a systematic literature review and meta-analysis. PLoS Negl Trop Dis. 2024;18:e0012254. https://doi.org/10.1371/journal.pntd.0011377.
28. Ogunlade ST, Meehan MT, Adekunle AI, Rojas DP, Adegboye OA, McBryde ES. A review: *Aedes*-borne arboviral infections, controls and *Wolbachia*-based strategies. Vaccines. 2021;9:32. https://doi.org/10.3390/vaccines9010032.
29. Gyawali N, Bradbury RS, Taylor-Robinson AW. The global spread of Zika virus: is public and media concern justified in regions currently unaffected? Infect Dis Poverty. 2016;5:37. https://doi.org/10.1186/s40249-016-0132-y.
30 Zheng S. The symptoms and treatment of Zika virus infection. E3S Web Conf. 2020;218:03048. https://doi.org/10.1051/e3sconf/202021803048.
31. Johansson MA, Mier-y-Teran-Romero L, Reefhuis J, Gilboa SM, Hills SL. Zika and the risk of microcephaly. N Engl J Med. 2016;375:1–4.
32. Silva NIO, Albery GF, Arruda MS, Oliveira GFG, Costa TA, de Mello ÉM, et al. Ecological drivers of sustained enzootic yellow fever virus transmission in Brazil, 2017–2021. PLoS Negl Trop Dis. 2023;17:e0011407.
33. Gianchecchi E, Cianchi V, Torelli A, Montomoli E. Yellow fever: origin, epidemiology, preventive strategies and future prospects. Vaccines. 2022;10:372. https://doi.org/10.3390/vaccines10030372.
34. Ferreira FC, Bastos Camacho LA, Villela DAM. Occurrence of yellow fever outbreaks in a partially vaccinated population: an analysis of the effective reproduction number. PLoS Negl Trop Dis. 2022;16:e0010741. https://doi.org/10.1371/journal.pntd.0010741.
35 Juan-Giner A, Hombach J. The life-long protective immunity of yellow fever vaccination: time to review? Lancet Glob Health. 2024;12:e352–3. https://doi.org/10.1016/S2214-109X(23)00594-6.
36. Garske T, Van Kerkhove MD, Yactayo S, Ronveaux O, Lewis RF, Staples JE, et al. Yellow fever in Africa: estimating the burden of disease and impact of mass vaccination from outbreak and serological data. PLoS Med. 2014;11:e1001638. https://doi.org/10.1371/journal.pmed.1001638.

37. Moore SM. The current burden of Japanese encephalitis and the estimated impacts of vaccination: combining estimates of the spatial distribution and transmission intensity of a zoonotic pathogen. PLoS Negl Trop Dis. 2021;15:e0009385. https://doi.org/10.1371/journal.pntd.0009385.
38. World Health Organization (WHO). Japanese encephalitis virus. [cited 2024 Jul 7]. Available from: https://www.who.int/news-room/fact-sheets/detail/japanese-encephalitis
39. Smithburn KC, Hughes TP, Burke AW, Paul JH. A neurotropic virus isolated from the blood of a native of Uganda. Am J Trop Med Hyg. 1940;20:411–22.
40 Chancey C, Grinev A, Volkova E, Rios M. The global ecology and epidemiology of West Nile virus. Biomed Res Int. 2015;2015:376230. https://doi.org/10.1155/2015/376230.
41. Schwarz ER, Long MT. Comparison of West Nile virus disease in humans and horses: exploiting similarities for enhancing syndromic surveillance. Viruses. 2023;15:1230. https://doi.org/10.3390/v15061230.
42 Troupin A, Colpitts TM. Overview of West Nile virus transmission and epidemiology. In: Colpitts TM, editor. West Nile virus: methods and protocols. New York: Springer; 2016. p. 15–8.
43 Heath CJ, Grossi-Soyster EN, Ndenga BA, Mutuku FM, Sahoo MK, Ngugi HN, et al. Evidence of transovarial transmission of Chikungunya and dengue viruses in field-caught mosquitoes in Kenya. PLoS Negl Trop Dis. 2020;14(6):e0008362.https://doi.org/10.1371/journal.pntd.0008362.
44. Fechter-Leggett E, Nelms BM, Barker CM, Reisen WK. West Nile virus cluster analysis and vertical transmission in *Culex pipiens* complex mosquitoes in Sacramento and Yolo Counties, California, 2011. J Vector Ecol. 2012;37:442–9. https://doi.org/10.1111/j.1948-7134.2012.00248.x.
45 Kaavya K, Tharakan J, Joshi CO, Aneesh EM. Role of vertically transmitted viral and bacterial endosymbionts of *Aedes* mosquitoes. Does paratransgenesis influence vector-borne disease control? Symbiosis. 2022;86:139–53. https://doi.org/10.1007/s13199-022-00836-1.
46 Louten J. Emerging and reemerging viral diseases. In: Louten J, editor. Essential human virology. Amsterdam: Elsevier; 2016. p. 291.
47. Lorenz C, Azevedo TS, Virginio F, Aguiar BS, Chiaravalloti-Neto F, Suesdek L. Impact of environmental factors on neglected emerging arboviral diseases. PLoS Negl Trop Dis. 2017;11:e0005959. https://doi.org/10.1371/journal.pntd.0005959.
48. Dong B, Khan L, Smith M, et al. Spatio-temporal dynamics of three diseases caused by *Aedes*-borne arboviruses in Mexico. Commun Med. 2022;2:134. https://doi.org/10.1038/s43856-022-00192-7.
49 Shocket MS, et al. Environmental drivers of vector-borne diseases. In: Drake JM, Bonsall M, Strand M, editors., et al., Population biology of vector-borne diseases. Oxford: Oxford Academic; 2020.
50 Lequime S, Lambrechts L. Vertical transmission of arboviruses in mosquitoes: a historical perspective. Infect Genet Evol. 2014;28:681–90. https://doi.org/10.1016/j.meegid.2014.07.025.
51. Ferreira-de-Lima VH, Lima-Camara TN. Natural vertical transmission of dengue virus in *Aedes aegypti* and *Aedes albopictus*: a systematic review. Parasit Vectors. 2018;11:77. https://doi.org/10.1186/s13071-018-2643-9.
52 Janjoter S, Kataria D, Yadav M, Dahiya N, Sehrawat N. Transovarial transmission of mosquito-borne viruses: a systematic review. Front Cell Infect Microbiol. 2024;13:1304938. https://doi.org/10.3389/fcimb.2023.1304938.
53. Favier C, Chalvet-Monfray K, Sabatier P, Lancelot R, Fontenille D, Dubois MA. Rift Valley fever in West Africa: the role of space in endemicity. Trop Med Int Health. 2006;11:1878–88. https://doi.org/10.1111/j.1365-3156.2006.01746.x.
54. Koolhof IS, Beeton N, Bettiol S, Charleston M, Firestone SM, Gibney K, et al. Testing the intrinsic mechanisms driving the dynamics of Ross River virus across Australia. PLoS Pathog. 2024. https://doi.org/10.1371/journal.ppat.1011944.
55. Hayes CG, Basit A, Bagar S, Akhter R. Vector competence of *Culex* tritaeniorhynchus (diptera: Culicidae) for west nile virus. J Med Entomol. 1980;17:172–7.
56. Baqar S, Hayes CG, Murphy JR, Watts DM. Vertical transmission of West Nile virus by *Culex* and *Aedes* species mosquitoes. Am J Trop Med Hyg. 1993;48:757–62.
57. Adams B, Boots M. How important is vertical transmission in mosquitoes for the persistence of dengue? Insights Math Model Epidemics. 2010;2:1-10. https://doi.org/10.1016/j.epidem.2010.01.001.
58. Gu W, Lampman R, Novak RJ. Problems in estimating mosquito infection rates using minimum infection rate. J Med Entomol. 2003;40:595-6. https://doi.org/10.1603/0022-2585-40.5.595.
59. Taghikhani R, Gumel AB. Mathematics of dengue transmission dynamics: roles of vector vertical transmission and temperature fluctuations. Infect Dis Model. 2018;3:266–92. https://doi.org/10.1016/j.idm.2018.09.003.
60 Chitnis N, Hyman JM, Manore CA. Modelling vertical transmission in vector-borne diseases with applications to RVFV. J Biol Dyn. 2013;7:11–40. https://doi.org/10.1080/17513758.2012.733427.
61. Yuan X, Lou Y, He D, Wang J, Gao D. A Zika endemic model for the contribution of multiple transmission routes. Bull Math Biol. 2021;83:1–28.
62. Abidemi A, Ahmad R, Aziz NAB. Assessing the roles of human movement and vector vertical transmission on dengue fever spread and control in connected patches: from modelling to simulation. Eur Phys J Plus. 2021;136:1192.
63. Abdullah S, Seadawy A, Jun W. New mathematical model of vertical transmission and cure of vector-borne diseases and its numerical simulation. Adv Differ Equ. 2018;2018:1-15. https://doi.org/10.1186/s13662-018-1516-z.
64. Alves LD, Lana RM, Coelho FC. A framework for weather-driven dengue virus transmission dynamics in different Brazilian regions. Int J Environ Res Public Health. 2021;18:9493. https://doi.org/10.3390/ijerph18189493.
65 Murillo D, Murillo A, Lee S. The role of vertical transmission in the control of dengue fever. Int J Environ Res Public Health. 2019;16:803. https://doi.org/10.3390/ijerph16050803.
66. Blayneh KW. Uniform persistence and backward bifurcation of vertically transmitted vector-borne diseases. Res Math. 2023;10:2264581. https://doi.org/10.1080/27684830.2023.2264581.
67. Aliyu AI, Inc M, Yusuf A, Baleanu D. A fractional model of vertical transmission and cure of vector-borne diseases pertaining to the Atangana-Baleanu fractional derivatives. Chaos Solitons Fractal. 2018;116:268–77. https://doi.org/10.1016/j.chaos.2018.09.043.
68. Fan G, Liu J, Van den Driessche P, Wu J, Zhu H. The impact of maturation delay of mosquitoes on the transmission of West Nile virus. Math Biosci. 2010;228:119–26. https://doi.org/10.1016/j.mbs.2010.04.004.
69. Wang FB, Wu R, Zhao XQ. A West Nile virus transmission model with periodic incubation periods. SIAM J Appl Dyn Syst. 2019;18:1498–535. https://doi.org/10.1137/18M1236162.
70. Wang Y, Li Y, Liu L, Liu X. A periodic Chikungunya model with virus mutation and transovarial transmission. Chaos Solitons Fractal. 2022;158:112002.
71. Cheng Q, Jing Q, Spear RC, Marshall JM, Yang Z, Gong P. Climate and the timing of imported cases as determinants of the dengue outbreak in Guangzhou, 2014: evidence from a mathematical model. PLoS Negl Trop Dis. 2016;10:e0004417. https://doi.org/10.1371/journal.pntd.0004417.
72. Cavalerie L, Charron MV, Ezanno P, Dommergues L, Zumbo B, Cardinale E. A stochastic model to study Rift Valley fever persistence with different seasonal patterns of vector abundance: new insights on the endemicity in the tropical island of Mayotte. PLoS ONE. 2015;10:e0130838. https://doi.org/10.1371/journal.pone.0130838.
73. Nuñez AI, Talavera S, Birnberg L, Rivas R, Pujol N, Verdún M, et al. Evidence of Zika virus horizontal and vertical transmission in *Aedes albopictus* from Spain but not infectious virus in saliva of the progeny. Emerg Microbe Infect. 2020;9:2236–44. https://doi.org/10.1080/22221751.2020.1830718.
74 Stephenson CJ, Coatsworth H, Kang S, Lednicky JA, Dinglasan RR. Transmission potential of Floridian *Aedes aegypti* mosquitoes for DENV virus serotype 4: Implications for estimating local dengue risk. mSphere. 2021;6:e00271-e321. https://doi.org/10.1128/mSphere.00271-21.
75. Bergren NA, Kading RC. The ecological significance and implications of transovarial transmission among the vector-borne bunyaviruses: a review. Insects. 2018;9:173. https://doi.org/10.3390/insects9040173.
76. Dahl E, Öborn L, Sjöberg V, Lundkvist Å, Hesson JC. Vertical transmission of Sindbis virus in *Culex* mosquitoes. Viruses. 2022;14:1915. https://doi.org/10.3390/v14091915.

77. Rudolf I, Betášová L, Blažejová H, et al. West Nile virus in overwintering mosquitoes, central Europe. Parasit Vectors. 2017;10:452. https://doi.org/10.1186/s13071-017-2399-7.
78. Bellini R, Medici A, Calzolari M, Bonilauri P, Cavrini F, Sambri V, et al. Impact of Chikungunya virus on *Aedes albopictus* females and possibility of vertical transmission using the actors of the 2007 outbreak in Italy. PLoS ONE. 2012;7:e28360.
79. Turell MJ. Virus-dependent mortality in Rift Valley fever, eastern equine encephalomyelitis, and chikungunya virus-inoculated mosquito (Diptera: Culicidae) larvae. J Med Entomol. 1992;29:792–5.
80. Mourya DT, Gokhale MD, Basu A, Barde PV, Sapkal GN, Padbidri VS, et al. Horizontal and vertical transmission of dengue virus type 2 in highly and lowly susceptible strains of *Aedes aegypti* mosquitoes. Acta Virol. 2001;45:67–72.
81. Joshi V, Mourya DT, Sharma RC. Persistence of dengue-3 virus through transovarial transmission passage in successive generations of *Aedes aegypti* mosquitoes. Am J Trop Med Hyg. 2002;67:158–61.
82. Satoto TBT, Umniyati S, Suardipa A, Sintorini M. Effects of temperature, relative humidity, and DEN-2 virus transovarial infection on viability of *Aedes aegypti*. Kesmas. 2013;7:331–6.
83. Sheela AM, Ghermandi A, Vineetha P, Sheeja RV, Justus J, Ajayakrishna K. Assessment of relation of land use characteristics with vector-borne diseases in tropical areas. Land Use Policy. 2017;63:369–80. https://doi.org/10.1016/j.landusepol.2017.01.047.
84. Edillo FE, Sarcos JR, Sayson SL. Natural vertical transmission of dengue viruses in *Aedes aegypti* in selected sites in Cebu City, Philippine. J Vector Ecol. 2015;40:282–91.
85. Zhang YM, Guo XX, Jiang SF, Li CX, Xing D, Zhang HD, et al. The potential vector competence and overwintering of West Nile virus in vector *Aedes albopictus* in China. Front Microbiol. 2022;13:888751.
86. Guo X, Zhao T, Dong Y, Lu B. Survival and replication of dengue-2 virus in diapausing eggs of *Aedes albopictus* (Diptera: Culicidae). J Med Entomol. 2007;44:492–7.
87. Freier JE, Rosen L. Vertical transmission of dengue viruses by mosquitoes of the *Aedes* scutellaris group. Am J Trop Med Hyg. 1987;37:640–7.
88. Rosen L, Shroyer DA, Tesh RB, Freier JE, Lien JC. Transovarial transmission of dengue viruses by mosquitoes: *Aedes albopictus* and *Aedes aegypti*. Am J Trop Med Hyg. 1983;32:1108–19.
89. Velandia-Romero ML, Olano VA, Coronel-Ruiz C, Cabezas L, Calderón-Peláez MA, Castellanos JE, et al. Dengue virus detection in *Aedes aegypti* larvae and pupae collected in rural areas of Anapoima, Cundinamarca, Colombia. Biomedica. 2017;37:193–200.
90. Mitchell CJ, Miller BR. Vertical transmission of dengue viruses by strains of *Aedes albopictus* recently introduced into Brazil. J Am Mosq Control Assoc. 1990;6:251–3.
91. Manuel M, Missé D, Pompon J. Highly efficient vertical transmission for Zika virus in *Aedes aegypti* after long extrinsic incubation time. Pathogens. 2020;9:366.
92. Zhu C, Jiang Y, Zhang Q, Gao J, Gu Z, Lan C, et al. Vertical transmission of zika virus by jiegao and mengding *Aedes aegypti* (Diptera: Culicidae) strains in Yunnan province in China. Vector Borne Zoonotic Dis. 2020;20:664–9.
93. Comeau G, Zinna RA, Scott T, Ernst K, Walker K, Carrière Y, et al. Vertical transmission of Zika virus in *Aedes aegypti* produces potentially infectious progeny. Am J Trop Med Hyg. 2020;103:876. https://doi.org/10.4269/ajtmh.19-0698.
94. Franklinos LH, Jones KE, Redding DW, Abubakar I. The effect of global change on mosquito-borne disease. Lancet Infect Dis. 2019;19:e302–12. https://doi.org/10.1016/S1473-3099(19)30161-6.
95. Cao B, Bai C, Wu K, La T, Su Y, Che L, et al. Tracing the future of epidemics: coincident niche distribution of host animals and disease incidence revealed climate-correlated risk shifts of main zoonotic diseases in China. Glob Chang Biol. 2023;29:3723–46. https://doi.org/10.1111/gcb.16708.
96 Bellone R, Failloux AB. The role of temperature in shaping mosquito-borne viruses transmission. Front Microbiol. 2020;11:584846. https://doi.org/10.3389/fmicb.2020.584846.
97 Mbaoma OC, Thomas SM, Beierkuhnlein C. Spatiotemporally explicit epidemic model for West Nile virus outbreak in Germany: an inversely calibrated approach. J Epidemiol Glob Health. 2024. https://doi.org/10.1007/s44197-024-00254-0.
98. Eisen L, Monaghan AJ, Lozano-Fuentes S, Steinhoff DF, Hayden MH, Bieringer PE. The impact of temperature on the bionomics of *Aedes* (Stegomyia) *aegypti*, with special reference to the cool geographic range margins. J Med Entomol. 2014;51:496–516. https://doi.org/10.1603/ME13214.
99 Ahmed T, Hyder MZ, Liaqat I, Scholz M. Climatic conditions: conventional and nanotechnology-based methods for the control of mosquito vectors causing human health issues. Int J Environ Res Public Health. 2019;16:3165. https://doi.org/10.3390/ijerph16173165.
100. Fay RL, Cruz-Loya M, Keyel AC, Price DC, Zink SD, Mordecai EA, et al. Population-specific thermal responses contribute to regional variability in arbovirus transmission with changing climates. iScience. 2024;27:109934. https://doi.org/10.1016/j.isci.2024.109934.
101. Diouf B, Gaye A, Diagne CT, Diallo M, Diallo D. Zika virus in southeastern Senegal: survival of the vectors and the virus during the dry season. BMC Infect Dis. 2020;20:1–9.
102. Rohani A, Azahary AA, Malinda M, Zurainee MN, Rozilawati H, Najdah WW, et al. Eco-virological survey of *Aedes* mosquito larvae in selected dengue outbreak areas in Malaysia. J Vector Borne Dis. 2014;51:327–32.
103. Golding MA, Noble SA, Khouri NK, Layne-Yarde RN, Ali I, Sandiford SL. Natural vertical transmission of dengue virus in Latin America and the Caribbean: highlighting its detection limitations and potential significance. Parasit Vectors. 2023;16:442. https://doi.org/10.1186/s13071-023-06043-1.
104. Coroian M, Silaghi C, Tews BA, Baltag ES, Marinov M, Alexe V, et al. Serological survey of mosquito-borne arboviruses in wild birds from important migratory hotspots in Romania. Pathogens. 2022;11:1270. https://doi.org/10.3390/pathogens11111270.
105. Thongrungkiat S, Maneekan P, Wasinpiyamongkol L, Prummongkol S. Prospective field study of transovarial dengue-virus transmission by two different forms of *Aedes aegypti* in an urban area of Bangkok, Thailand. J Vector Ecol. 2011;36:147–52. https://doi.org/10.1111/j.1948-7134.2011.00151.x.
106. Teixeira AF, de Brito BB, Correia TML, Viana AIS, Carvalho JC, da Silva FAF, et al. Simultaneous circulation of Zika, Dengue, and Chikungunya viruses and their vertical co-transmission among *Aedes aegypti*. Acta Trop. 2021;215:105819. https://doi.org/10.1016/j.actatropica.2020.105819.
107. Granger Neto HP, Rocha CV, Correia TML, Silva NMPD, Chaves BA, Secundino NFC, et al. Natural vertical co-transmission of Dengue virus and Chikungunya virus from *Aedes aegypti* in Brumado, Bahia, Brazil. Rev Soc Bras Med Trop. 2022;55:e0427-2021. https://doi.org/10.1590/0037-8682-0427-2021.
108 Guimarães NR, Cezar IS, Ramos KS, Meira CS. Panorama epidemiológico da dengue na Bahia: Uma década de dados (2014–2023). Res Soc Dev. 2024;13:e9513846351.
109. Cecílio AB, Campanelli ES, Souza KPR, Figueiredo LB, Resende MC. Transmissão vertical natural de *Stegomyia albopicta como* vetor de dengue no Brasil. Braz J Biol. 2009;69:123–7. https://doi.org/10.1590/S1519-69842009000100015.
110. Pessanha JEM, Caiaffa WT, Cecilio AB, Iani FCDM, Araujo SC, Nascimento JC, et al. Cocirculation of two dengue virus serotypes in individual and pooled samples of *Aedes aegypti* and *Aedes albopictus* larvae. Rev Soc Bras Med Trop. 2011;44:103–5. https://doi.org/10.1590/S0037-86822011000100023.

Publisher's Note

Springer Nature remains neutral with regard to jurisdictional claims in published maps and institutional affiliations.
